# Synthesis and Antiproliferative Activity of New Cyclodiprenyl Phenols against Select Cancer Cell Lines

**DOI:** 10.3390/molecules23092323

**Published:** 2018-09-12

**Authors:** Bastián Said, Iván Montenegro, Manuel Valenzuela, Yusser Olguín, Nelson Caro, Enrique Werner, Patricio Godoy, Joan Villena, Alejandro Madrid

**Affiliations:** 1 Departamento de Química, Universidad Técnica Federico Santa María, Av. Santa María 6400, Vitacura 7630000, Santiago, Chile; bastian.said@usm.cl; 2 Escuela de Obstetricia y Puericultura, Facultad de medicina, Campus de la Salud, Universidad de Valparaíso, Angamos 655, Reñaca, Viña del Mar 2520000, Chile; ivan.montenegro@uv.cl; 3 Laboratorio de Microbiología Celular, Instituto de Investigación e Innovación en Salud, Facultad de Ciencias de la Salud, Universidad Central de Chile, Santiago 8320000, Chile; manuel.valenzuela@ucentral.cl; 4 Center for Integrative Medicine and Innovative Science (CIMIS), Facultad de Medicina, Universidad Andrés Bello, Santiago 8320000, Chile; yusser.olguin@unab.cl; 5 Centro de Investigación Australbiotech, Universidad Santo Tomás, Avda. Ejército 146, Santiago 8320000, Chile; ncaro@australbiotech.cl; 6 Departamento De Ciencias Básicas, Campus Fernando May Universidad del Biobío, Avda. Andrés Bello s/n casilla 447, Chillán 3780000, Chile; ewerner@ubiobio.cl; 7 Instituto de Microbiología Clínica, Facultad de Medicina, Universidad Austral de Chile, Los Laureles s/n, Isla Teja, Valdivia 5090000, Chile; patricio.godoy@uach.cl; 8 Centro de Investigaciones Biomedicas (CIB), Facultad de Medicina, Campus de la Salud, Universidad de Valparaíso, Angamos 655, Reñaca, Viña del Mar 2520000, Chile; 9 Laboratorio de Productos Naturales y Síntesis Orgánica, Departamento de Química, Facultad de Ciencias Naturales y Exactas, Universidad de Playa Ancha, Avda. Leopoldo Carvallo 270, Playa Ancha, Valparaíso 2340000, Chile

**Keywords:** perillyl alcohol, synthesis, cyclodiprenyl phenols, antiproliferative agents

## Abstract

Six new cyclodiprenyl phenols were synthesized by direct coupling of perillyl alcohol and the appropriate phenol. Their structures were established by IR, HRMS and mainly NMR. Three human cancer cell lines—breast (MCF-7), prostate (PC-3) and colon (HT-29)—were used in antiproliferative assays, with daunorubicin and dunnione as positive controls. Results described in the article suggest that dihydroxylated compounds **2**–**4** and monohydroxylated compound **5** display selectivity against cancer cell lines, cytotoxicity, apoptosis induction, and mitochondrial membrane impairment capacity. Compound **2** was identified as the most effective of the series by displaying against all cancer cell lines a cytotoxicity close to dunnione antineoplastic agent, suggesting that the cyclodiprenyl phenols from perillyl alcohol deserve more extensive investigation of their potential medicinal applications.

## 1. Introduction

Many natural products of mixed biosynthesis, such as meroterpenes, have been reported from both terrestrial and marine sources [1]. In this context, recent years have seen major advances in research and development concerning meroterpenes whose antiproliferative activity appears promising for the treatment of cancer [2,3,4]. These are mostly hydroquinones with a terpenoid portion ranging in size from one to nine isoprene units. In particular, sesquiterpene hydroquinones from sponges such as arenarol, fulvanin-2, yahazunol, avinosol and avarol offer promising opportunities for the development of new antitumor agents [2]. Based on these premises, the potential of meroterpenes is of great interest; however, low yields of these compounds have traditionally been obtained from natural sources [5,6,7,8]. For these reasons, research efforts to chemically synthesize these compounds, their structural analogs, and their derivatives have intensified in recent decades [9,10,11,12]. However, little has been done on the synthesis and biological evaluation of hybrid molecules combining cyclic monoterpenes and synthetic phenols [13,14,15]. One of the best known cyclic monoterpenes is perillyl alcohol, a small lipophilic allylic alcohol found predominantly in essential oils from *Perilla frutescens*, cherries, cranberries, lavender, celery seed and spearmint [16,17], which exhibit chemopreventive and cytotoxic activity against a wide variety of cancer cell lines [18,19,20,21,22,23,24]. Additionally, the present findings suggest that perillyl alcohol may be used as a prototype for the prevention of ethanolic liver injury and in therapy of patients with malignant brain tumors [25,26]. These facts motivated us to accomplish the synthesis of novel cyclodiprenyl phenols from perillyl alcohol **1** and different phenol moieties. In terms of application, our final aim was the evaluation of their biological activity as potential antiproliferative agents against a panel of cancer cell lines.

## 2. Results and Discussion

### 2.1. Synthesis of Cyclodiprenyl Phenols

**Scheme 1:**
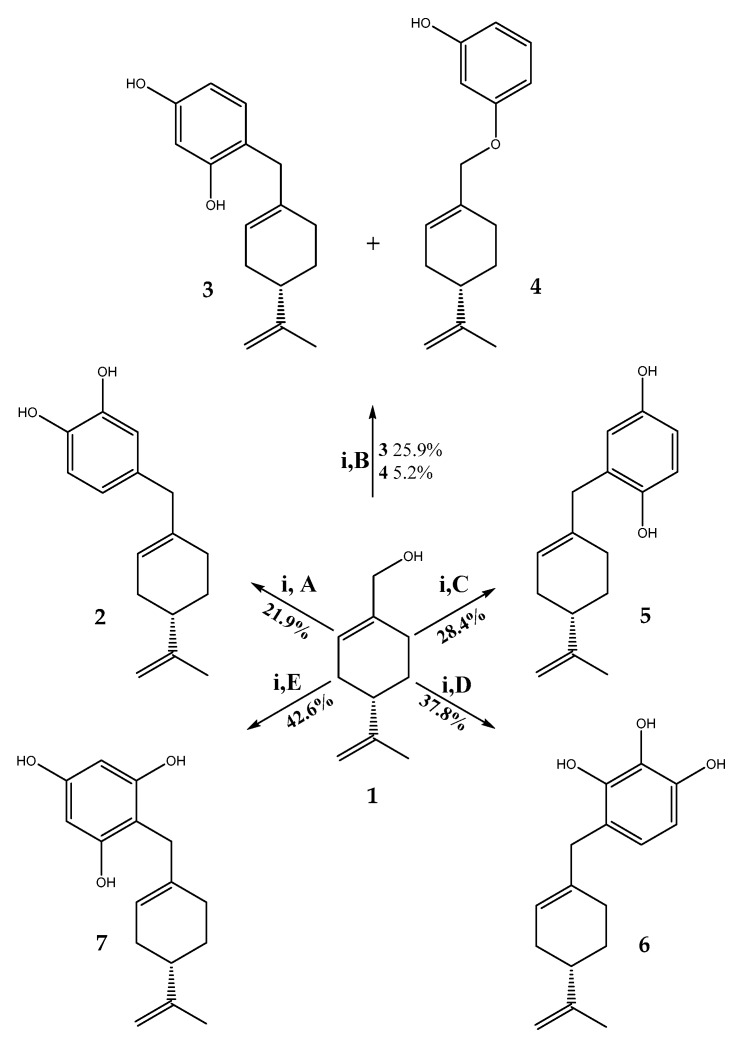
Synthesis of cyclodiprenyl phenols **2**–**7**. *Reagents and conditions*: (**i**) BF_3_·OEt_2_, CH_3_CN, N_2_ at r.t. for 48 h.; (**A**) pyrocatechol; (**B**) resorcinol; (**C**) hydroquinone; (**D**) pyrogallol; (**E**) phloroglucinol.

The new cyclodiprenyl phenols **2**–**7** were synthesized from perillyl alcohol **1** by alkylation with the corresponding phenol in acetonitrile in the presence of boron trifluoride diethyl etherate as catalyst (Scheme 1) [27].

Afterwards, novel cyclodiprenyl phenols were obtained in moderate yields. Nevertheless, compound **1** reacts with resorcinol in the presence of boron trifluoride etherate to produce compound **3** as a major product via Friedel–Crafts alkylation, and compound **4** as a minor product via retro-Friedel–Crafts alkylation, due to this Lewis acid mediated coupling is reversible (Scheme 1) [28]. Since no other minor products resulted from reaction of perillyl alcohol **1** with other phenols under the same conditions, such byproducts could thus be recycled to contribute towards the yield of the desired product.

The structure of each new derivatives **2**–**7** was unambiguously assigned by IR spectroscopy, ^1^H-/^13^C-NMR data, and confirmed by high resolution mass spectrometry. The ^1^H- and ^13^C-NMR data for the derivatives of perillyl alcohol were nearly identical in the aliphatic region of the spectra [29,30], and the substitution position of perilic unit in aromatic ring was established by two-dimensional (2D) HMBC correlations (see Supplementary Materials).

**Figure 1:**
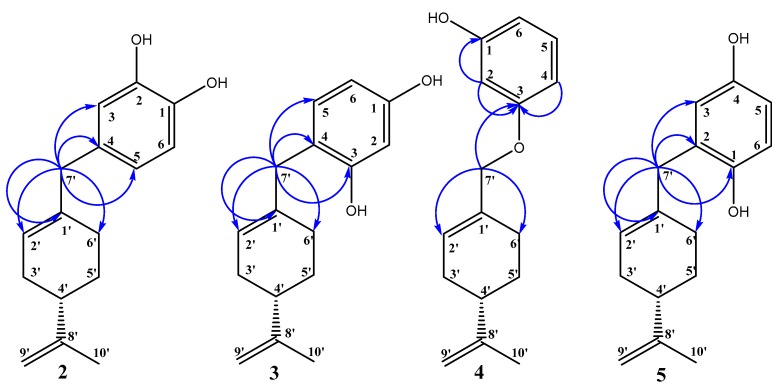
Most important correlations 2D ^1^H–^13^C HMBC of compounds **2**–**5**.

For the synthesis of compound **2**, pyrocatechol and perillyl alcohol were used. In the ^1^H-NMR spectrum, the signals at *δ*_H_ = 6.76 (d, *J* = 8.0 Hz, 1H, H-6), 6.68 (s, 1H, H-3) and 6.60 (d, *J* = 8.0 Hz, 1H, H-5) ppm confirm the presence of a trisubstituted aromatic system. In the 2D HMBC spectrum, the signal at *δ*_H_ = 3.27 ppm (s, 2H, H-7′) showed *^3^J*_H-C_ coupling with C-5 and C-3 (*δ*_C_ = 121.2 and 115.1 ppm, respectively) and ^2^*J*_H-C_ coupling with C-4 (*δ*_C_ = 137.1 ppm), confirming that perilic unit was substituted in the *meta* position respect to the hydroxyl group on the aromatic core, while other HMBC correlations are shown in Figure 1.

On the other hand, the direct alkylation between resorcinol and **1** produced compound **3**. The structure and pattern of the aromatic monosubstitution of this compound were established from NMR data spectra. In ^1^H-NMR spectrum, the signals at *δ*_H_ = 6.90 ppm (d, *J* = 8.4 Hz, 1H, H-5) and *δ*_H_ = 6.34 ppm (m, 2H, H-2 and H-6) confirm the presence of a trisubstituted aromatic system. The *ortho* position of the perillic unit substitution in the aromatic core was determined from the 2D HMBC spectrum, the signal at *δ*_H_ = 3.27 ppm (s, 2H, H-7′) showed heteronuclear ^3^*J*_H-C_ coupling with C-3 (*δ*_C_ = 156.0 ppm) and C-5 (*δ*_C_ = 131.2 ppm) and ^2^*J*_H–C_ coupling C-4 (*δ*_C_ = 117.0 ppm).

For compound **4**, the signal at *δ*_H_ = 4.36 ppm (s, 2H, H-7′) ascribed to O-CH_2_ protons of an alkoxy chain linked to the aromatic system is observed. This is characteristic of allylic chains on aromatic rings, resulting from the alkylation reaction. These data also were corroborated by 2D HMBC correlations, where H-7′ showed heteronuclear *^3^J* correlations with C-3 (*δ*_C_ = 160.4 ppm), C-2′ (*δ*_C_ = 125.1 ppm) and heteronuclear *^2^J* correlations were also observed with C-1′ (*δ*_C_ = 133.4 ppm) (Figure 1).

Our next goal was the preparation of compound **5**, using **1** and hydroquinone as the starting materials. In the ^1^H-NMR spectrum, the three aromatic signals at *δ*_H_ = 6.70 (d, *J* = 8.3 Hz, 1H, H-6), 6.60 (d, *J* = 8.3 Hz, 1H, H-5) and *δ*_H_ = 6.58 (s, H, H-3) ppm show a typical ABC pattern (1,2,4. tri-substituted aromatic system), the spectrum in this region being identical with that of prenylhydroquinone and geranylhydroquinone [31]. The HMBC correlations are shown in Figure 1.

**Figure 2:**
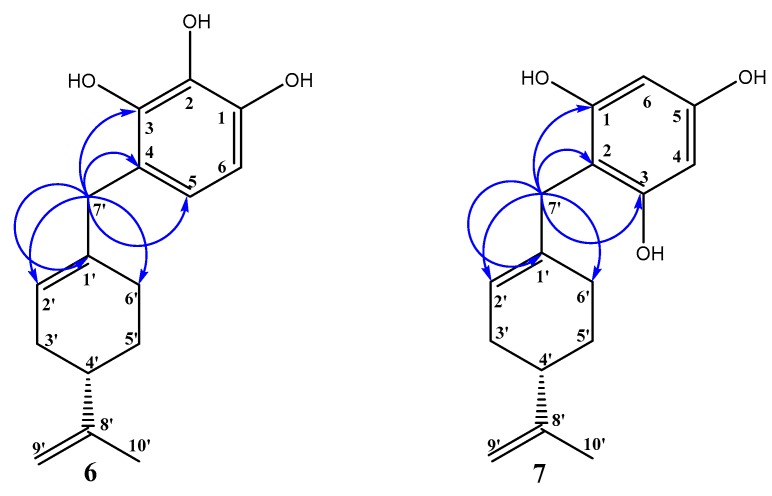
Most important correlations 2D ^1^H–^13^C HMBC of compounds **6** and **7**.

The following step consisted in the preparation of compound **6**. The structure of **6** was established by NMR, where aromatic signals at *δ*_H_ = 6.49 (d, *J* = 8.3 Hz, 1H) and 6.43 (d, *J*=8.3 Hz, 1H) were observed as two doublets for hydrogens H-5 and H-6 respectively, confirming the aromatic substitution. Additionally, in the HMBC spectrum, the signal at *δ*_H_ = 3.25 ppm assigned to H-7′ (s, 2H) shows ^3^*J*_H-C_ coupling with C-3 (*δ*_C_ = 143.0 ppm), C-5 (*δ*_C_ = 121.2 ppm) and C-6 (*δ*_C_ = 122.6 ppm) and ^2^*J*_H-C_ coupling with C-1′ and C-4 (*δ*_C_ = 137.4 ppm and 117.6 ppm respectively). These HMBC correlations are shown in Figure 2.

Finally, the synthesis of compound **7** was developed by a direct alkylation reaction between phloroglucinol and **1**. The determination of structure of **7** was mainly established by the NMR aromatic signal at *δ*_H_ = 5.98 ppm, where a singlet for two hydrogens (H-4 and H-6) was observed, confirming the unique possibility of aromatic monosubstitution. Additionally, the signal at *δ*_H_ = 3.29 ppm to assigned at H-7′ (d, *J* = 7.0 Hz, 2H) shows ^3^*J*_H-C_ coupling with C-1 and C-3 (*δ*_C_ = 156.3 ppm) and ^2^*J*_H-C_ coupling with C-2 (*δ*_C_ = 103.7 ppm), these HMBC correlations are shown in Figure 2.

### 2.2. In Vitro Activities

The natural compound **1** and meroterpenes **2**–**7** were evaluated for their in vitro cytotoxic activity on a panel of three human cancer cell lines—MCF-7 (breast), PC-3 (prostate) and HT-29 (colon)—and two human non-tumoral cell lines, human dermal fibroblasts (HDF) and colon epithelial cells (CoN) using the conventional sulforhodamine dye assay. The results obtained from these assays are shown in Table 1.

In this study, we evaluated the anti-cancer activity of a new cyclodiprenyl phenols against human prostate cancer cells (PC-3), reast B (MCF-7) and colon (HT-29) cancer cells. Cell viability analysis of cyclodiprenyl phenols indicated that compound derivatives showed more pronounced anti-proliferative activity than **1**. According to the IC_50_ values summarized in Table 1, it is evident that the alkylation of phenols by perillyl alcohol, as in derivatives **2**, **3**, **4** and **5** induces a remarkable increase of the cytotoxic activity in all the evaluated cell lines, compared to compound **1**. However, compounds **2** and **5** inhibit cellular viability on prostate cancer cells when compared with dunnione. Nevertheless, the compounds **6** and **7** did not affect the viability of the cells lines studied. Among the members of this series, compound **2** showed the highest cytotoxic activity against all cancer cell lines, without affecting non-tumoral cells. This great cytotoxicity can be influenced by the position of the perillic fragment with respect to the catechol system [32,33,34]. Phenolic compounds are the subject of intense scientific research because of the way they work to prevent or lower the risk of various cancers. Cancers caused or induced by free radicals can be effectively scavenged by polyphenols. We conclude on the basis of bond dissociation enthalpies (BDE) that the relative activity position of OH in the benzene ring is very important. According to literature, two postulates are proposed [35]:

(i) The position of OH′s is very determinant for lower BDE, but not the number of OH′s.
(ii) Increasing the number of OH′s in the vicinal (*ortho*) position, that is, more intramolecular hydrogen bond, decreases the BDE, but increasing the number of OH′s in the *meta* position has little impact on BDEs compared with a single OH group.

The empirical evidence shows that when the chain is in the *meta* position with respect to the OH group (compound **5**) the molecules obtained have a greater cytotoxic activity and the activity increases when the molecule presents an OH in the vicinal *ortho* position (compound **2**) in comparison with the molecules (compound **3**, **4**, **6**, and **7**) that presented the chain in the position *ortho* to the OH group.

Since compounds **2**–**5** had strong inhibitory effects on various cancer cell line growth and certain selectivity in relation to non-tumoral cells, we decided to study the effect of these compounds. To elucidate whether compounds **2**–**5** reduced the cell viability of MCF-7, PC-3 and HDF cells by inducing apoptosis as was previously described for perillyl alcohol [21], the cells treated with compounds **2**–**5** for 48 h were stained with Hoestch 33342 for 30 min. Condensed and/or fragmented nuclei, as an apoptotic characteristic, were observed under a fluorescence microscope (200×) and quantified in MCF-7, PC-3 and HDF cells, as shown in Table 2. Exposure to compounds **2**–**4** significantly affected the condensation and/or fragmentation nuclei in the treated cells versus control cells. The data indicates that compounds **2**–**4** induce changes in the morphology of the nuclei, which are related to an apoptotic cell death cycle [36]. On other hand, treatment with compound **5** had no effect in the morphology of the nuclei, suggesting that the cytotoxicity induced by this compound is not associated to an apoptotic cell death pathway. The analyzed compounds have no effect in nuclear morphology of HDF cells correlating with cytotoxicity data (Table 1) and suggesting a selective effect of compounds **2**–**4**.

Mitochondria are important organelles of the apoptosis execution machinery, which includes pro-apoptotic events involving decreased mitochondrial membrane potential (Δψm), release of cytochrome C and activation of caspase cascade [37,38]. Mitochondria play an important role in the cell death fate by serving as a convergent center of apoptotic signals originated from both the extrinsic and intrinsic pathways [39]. The changes induced in the mitochondria membrane potential have been previously reported to represent a determinant in the execution of cell death [40]. We analyzed the possible changes induced by the studied compounds in the mitochondrial membrane potential, using rhodamine 123 to track down changes in mitochondrial function, since fluorescence of the dye decreases as mitochondrial membrane potential is lost [41]. As shown in Table 3, treatment with compounds **2**–**4** (25 μM) significantly increased the percentage of cells without rhodamine 123 (* *p* < 0.05) in MCF-7 and PC-3 cell lines. Thus, compounds **2**–**4** induced loss of mitochondrial membrane potential correlated well with cytotoxic activity and apoptotic nuclear morphology (see Table 1 and Table 2).

**Figure 3:**
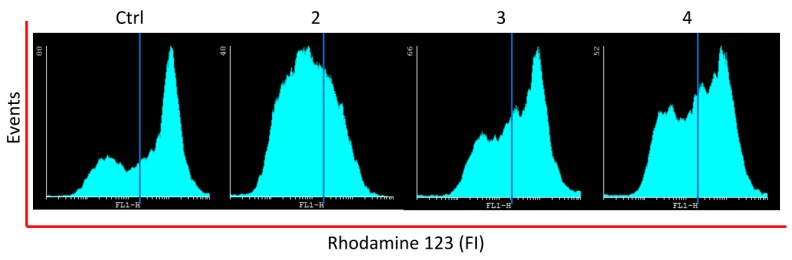
Effect of treatment with compounds **2**–**4** in mitochondrial membrane permeability in MCF-7 cell line was analyzed by flow cytometry. Cells were treated with compounds (25 µM), posteriorly stained with rhodamine 123 and then analyzed by flow cytometry. Representative histogram showing changes of mitochondrial membrane permeability.

In addition, a representative histogram showing changes of mitochondrial membrane permeability in MCF-7 cells is presented in Figure 3.

Aromatic systems induce loss of mitochondrial membrane potential in breast cancer cells. As shown in Figure 3, we observed that MCF-7 cancer cell when treated with compound **2** showed a strong loss of mitochondrial permeability effect. This decrease was significative in both the cell lines. Finally, the decrease of mitochondrial membrane potential is associated to the release of apoptogenic factors, such as cytochrome c and activation of caspases [42]. Next we investigated the effects of compounds **2**–**4** on caspase activity. As shown in Table 4, the caspase activation is higher in cells exposed to compounds **2**–**4** than in control cells in both cell lines. That is, compounds **2**–**4** increase caspases activity in cancer cells corroborating the cytotoxicity data and indicating that these compounds induced apoptotic cell death in the cell lines studied.

**Figure 4:**
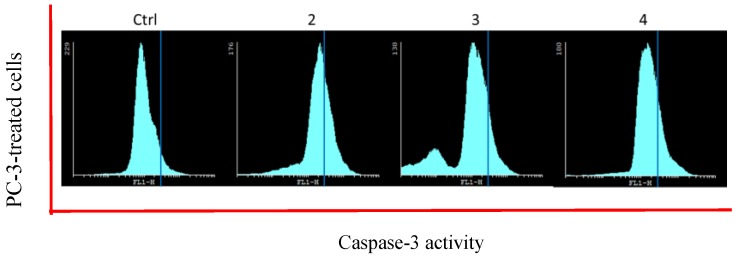
Effect of compounds **2**–**4** on caspases activity in PC-3 cell line was analyzed by flow cytometry. Cells were treated with compounds (25 µM), posteriorly stained with CaspACE™ FITC-VAD-FMK and then analyzed by flow cytometry. Representative histogram showing changes on caspases activity.

Additionally, a representative histogram showing changes on caspases activity in PC-3-treated cells is illustrated in Figure 4.

The caspase cascade is central to this process, as these cysteine proteases are cleaved from their proenzyme forms in response to proapoptotic stimuli. In particular, the cleavage of procaspase-3 to caspase-3 represents a critical node in apoptosis, as this executioner caspase catalyzes the hydrolysis of hundreds of protein substrates, leading to cell death [43]. We analyzed the effect of treatment with compound **2**, **3** and **4** on caspase-3 activation in the different cell lines. As shown in Figure 4, the activation of caspase-3 in cells exposed to compounds assayed is increased versus control-treated cells (1% ethanol). The three compounds exhibited potential as pro-apoptotic agents, with the highest activation observed for **2** (32.4 ± 4.0 and 23.4 ± 3.0-fold caspase-3 activity increase in comparison to the control) in MCF-7 and PC-3 cells, respectively. Similarly **4** caused apoptosis of a slightly lower scope, with a 21.4  ±  2.9 and 19.1  ±  2.8-fold caspase-3 activity in MCF-7 and PC-3 cells, respectively, while **3** has a moderate effect.

On the other hand, the results obtained confirm the potent cytotoxic activity of pyrocatechol and hydroquinone over resorcinol-derived systems against cancer cells is mediated by reactive oxygen species production via one-electron-based redox cycling [44,45]. However, upon administration in a model in vivo, these compounds undergo two main metabolic pathways, oxidation and conjugation. Oxidation generally leads to electrophilic quinone metabolites able to covalently bind proteins or DNA. In contrast, the conjugation reactions, sulfation, methylation, and glucuronidation, catalyzed by the respective enzymes, are effective detoxication mechanisms and allow the excretion of the conjugates into bile and urine [46,47]. Therefore, it is important to determine in future studies the factors, especially the structural features, which guide to this type of hydroxylated compounds toward different metabolic pathways and govern their biotransformation. In conclusion, the new cyclodiprenyl phenols were found to be highly promising potent antiproliferative agents and the presented data support their candidacy for further studies as a novel class of potential anticancer agents.

## 3. Materials and Methods

### 3.1. General Information

(*S*)-Perillyl alcohol, pyrocatechol, resorcinol, hydroquinone, pyrogallol, phloroglucinol and the others chemicals used were of reagent grade and were obtained from Aldrich (St. Louis, MO, USA). The reaction progress was monitored by thin layer chromatography on silica gel 60 F-254 (Merck, Darmstadt, Germany), and components were visualized by a VL-4LC UV lamp (Vilber Lournat, Collégien, France). Purification by flash chromatography was performed on silica gel 60 (particle size 0.032–0.063 mm) also from Merck and recrystallization. Melting points were measured on a SMP3 apparatus (Stuart-Scientific, Staffordshire, UK). FT-IR spectra were recorded on Buck Scientific M500 instrument (Buck Scientific Instrument, East Norwalk, CT, USA). NMR spectra were recorded at room temperature in solution on a 400 MHz Avance instrument (Bruker, Rheinstetten, Germany). HRMS spectra were recorded on a MAT 95 XL mass spectrometer (Thermo Finnigan, Bremen, Germany).

### 3.2. General Procedure for Obtaining Derivatives

In a round bottom flask BF_3_·OEt_2_ (0.3 mL, 2.43 mmol) was gradually added at room temperature to a solution of perillyl alcohol (**1**, 2.08 mmol) and different phenols (2.29 mmol) in dry acetonitrile (10 mL). The mixture was stirred at room temperature under a nitrogen atmosphere for 48 h, when the completion of the reaction was verified by TLC. The mixture was poured onto crushed ice (5 g). The two phases were separated and the water phase was extracted with diethyl ether (3 × 30 mL). The combined organic phases were washed with 5% NaHCO_3_ (15 mL), dried and the solvent was evaporated. The crude product was subjected to column chromatography (silica gel, *n*-hexane/ethyl acetate mixtures of increasing polarity) which provided the target compounds **2**–**7**. The % purity of compounds **2**–**7** were confirmed by analytical HPLC (compound **2**—98%, compound **3**—94%, compound **4**—95%, compound **5**—98%, compound **6**—96%, and compound **7**—97%).

*4-{[(4S)-4-Isopropenylcyclohex-1-en-1-yl]methyl}benzene-1,2-diol* (**2**). Pale yellow viscous oil. Yield: 21.9%. IR υ_max_ (KBr) cm^−1^: 3350 (O-H), 2920 (C-H), 1643 (C=C), 1515 (C=C), 1435 (C=C), 1276 (C-H). ^1^H-NMR (400.1 MHz, CDCl_3_): 6.76 (d, *J* = 8.0 Hz, 1H, H-6); 6.68 (s, 1H, H-3); 6.60 (d, *J* = 8.0 Hz, 1H, H-5); 5.69 (b.s., 1H, OH); 5.45 (s, 1H, H-2′); 4.69 (s, 2H, H-9′); 3.76 (b.s., 1H, OH); 3.14 (s, 2H, H-7′); 2.14 (m, 2H, H-3_β_′ and H-4′); 2.02 (m, 3H, H-3_α_′ and H-6′); 1.85 (m, 1H, H-5_β_′); 1.74 (s, 3H, H-10′); 1.47 (m, 1H, H-5_α_′). ^13^C-NMR (100.6 MHz, CDCl_3_): 150.1 (C-8′); 143.3 (C-1); 141.6 (C-2); 137.1 (C-1′); 133.4 (C-4); 122.2 (C-2′); 121.2 (C-5); 115.7 (C-6); 115.1 (C-3); 108.4 (C-9′); 43.5 (C-7′); 41.1 (C-4′); 30.5 (C-3′); 28.4 (C-5′); 27.8 (C-6′); 20.7 (C-10′). HRMS: M + H ion *m*/*z* 245.1543 (calcd. for C_16_H_21_O_2_, 245.1542).

*4-{[(4S)-4-Isopropenylcyclohex-1-en-1-yl]methyl}benzene-1,3-diol* (**3**). Orange solid. Yield: 25.9%. m.p. 77-78 °C. IR υ_max_ (KBr) cm^−1^: 3296 (O-H), 2921 (C-H), 1606 (C=C), 1517 (C=C), 1456 (C=C), 1163 (C-H). ^1^H-NMR (400.1 MHz, CDCl_3_): 6.90 (d, *J* = 8.4 Hz, 1H, H-5); 6.34 (m, 2H, H-2 and H-6); 5.41 (s, 1H, OH); 4.71 (m, 3H, OH and H-9′); 3.27 (s, 2H, H-7′); 2.16 (m, 2H, H-3_β_′ and H-4′); 1.98 (m, 3H, H-3_α_′ and H-6′); 1.78 (m, 1H, H-5_β_′); 1.73 (s, 3H, H-10′); 1.47 (m, 1H, H-5_α_′). ^13^C-NMR (100.6 MHz, CDCl_3_): 156.0 (C-3); 155.5 (C-1); 149.6 (C-8′); 136.9 (C-1′); 131.5 (C-5); 123.2 (C-2′); 117.0 (C-4); 108.7 (C-9′); 107.5 (C-6); 103.3 (C-2); 40.9 (C-4′); 39.3 (C-7′); 30.6 (C-3′); 28.3 (C-5′); 27.5 (C-6′); 20.7 (C-10′). HRMS: M + H ion *m*/*z* 245.1546 (calcd. for C_16_H_21_O_2_, 245.1542).

*3-{[(4S)-4-Isopropenylcyclohex-1-en-1-yl]methoxy}phenol* (**4**). Pale orange viscous oil. Yield: 5.2%. IR υ_max_ (KBr) cm^−1^: 3384 (O-H), 2925 (C-H), 1648(C=C), 1458 (C=C), 1382 (C-H), 1199 (Ar-O-R). ^1^H-NMR (400.1 MHz, CDCl_3_): 7.11 (t, *J* = 8.0 Hz, 1H, H-5); 6.50 (d, *J* = 7.6 Hz, 1H, H-4); 6.42 (m, 2H, H-2 and H-6); 5.80 (b.s, 1H, OH); 4.73 (m, 3H, OH and H-9′); 4.36 (s, 2H, H-7′); 2.18 (m, 2H, H-3_β_′ and H-4′); 1.99 (m, 3H, H-3_α_′ and H-6′); 1.87 (m, 1H, H-5_β_′); 1.75 (m, 4H, H-10′ and H-5_α_′). ^13^C-NMR (100.6 MHz, CDCl_3_): 160.7 (C-3); 156.3 (C-1); 149.7 (C-8′); 133.4 (C-1′); 130.0 (C-5); 125.1 (C-2′); 108.7 (C-9′); 107.6 (C-4); 107.3 (C-6); 102.3 (C-2); 72.3 (C-7′); 40.9 (C-4′); 30.5 (C-3′); 27.3 (C-5′); 26.3 (C-6′); 20.7 (C-10′). HRMS: M + H ion *m*/*z* 245.1544 (calcd. for C_16_H_21_O_2_, 245.1542).

*2-{[(4S)-4-Isopropenylcyclohex-1-en-1-yl]methyl}benzene-1,4-diol* (**5**). Pale yellow viscous oil. Yield: 28.4%. IR υ_max_ (KBr) cm^−1^: 3364 (O-H), 2922 (C-H), 1643 (C=C), 1503 (C=C), 1453 (C=C), 1199 (C-H). ^1^H-NMR (400.1 MHz, CDCl_3_): 6.70 (d, *J* = 8.3 Hz, 1H, H-6); 6.60 (d, *J* = 8.3 Hz, 1H, H-5); 6.58 (s, H, H-3); 5.63 (b.s, 1H, H-2); 4.99 (s, 1H, OH); 4.71 (s, 1H, H-9_β_′); 4.69 (s, 1H, H-9_α_′); 4.65 (s, 1H, OH); 3.26 (s, 2H, H-7′); 2.16 (m, 2H, H-3_β_′ and H-4′); 1.98 (m, 3H, H-3_α_′ and H-6′); 1.80 (m, 1H, H-5_β_′); 1.72 (s, 3H, H-10′); 1.47 (m, 1H, H-5_α_′). ^13^C-NMR (100.6 MHz, CDCl_3_): 149.6 (C-4); 149.3 (C-1 and C-8′); 136.2 (C-1′); 126.2 (C-2); 123.3 (C-2′); 117.5 (C-6); 114.2 (C-3); 114.0 (C-5); 108.7 (C-9′); 40.9 (C-4′); 39.7 (C-7′); 30.7 (C-3′); 28.5 (C-5′); 27.6 (C-6′); 20.7 (C-10′). HRMS: M + H ion *m*/*z* 245.1540 (calcd. for C_16_H_21_O_2_, 245.1542).

*4-{[(4S)-4-Isopropenylcyclohex-1-en-1-yl]methyl}benzene-1,2,3-triol* (**6**). Yellow viscous oil. Yield: 37.8%. IR υ_max_ (KBr) cm^−1^: 3375 (O-H), 2921 (C-H), 1627 (C=C), 1458 (C=C), 1367 (C-H), 1221 (C-H). ^1^H-NMR (400.1 MHz, CDCl_3_): 6.49 (d, *J* = 8.3 Hz, 1H, H-5); 6.43 (d, *J* = 8.3 Hz, 1H, H-6); 5.60 (s, 1H, H-2); 4.76 (s, 1H, H-9_β_′); 4.75 (s, 1H, H-9_α_′); 3.25 (s, 2H, H-7′); 2.11 (m, 2H, H-3_β_′ and H-4′); 1.98 (m, 3H, H-3_α_′ and H-6′); 1.89 (m, 1H, H-5_β_′); 1.71(s, 3H, H-10′); 1.51 (m, 1H, H-5_α_′). ^13^C-NMR (100.6 MHz, CDCl_3_): 150.0 (C-8′); 143.0 (C-3); 142.7 (C-1); 137.4 (C-1′); 131.9 (C-2); 122.6 (C-2′); 121.4 (C-5); 117.6 (C-4); 108.2 (C-9′); 107.4 (C-6); 44.9 (C-4′); 38.9 (C-7′); 29.7 (C-3′); 28.9 (C-5′); 27.3 (C-6′); 21.0 (C-10′). HRMS: M + H ion *m*/*z* 261.1490 (calcd. for C_16_H_21_O_3_, 261.1491).

*2-{[(4S)-4-Isopropenylcyclohex-1-en-1-yl]methyl}benzene-1,3,5-triol* (**7**). Brown solid. Yield: 42.6%. m.p. 144–146 °C. IR υ_max_ (KBr) cm^−1^: 3442 (O-H), 2923(C-H), 1632 (C=C), 1463 (C=C), 1223 (C-H). ^1^H-NMR (400.1 MHz, CDCl_3_): 6.02 (b.s, 2H, OH); 5.98 (s, 2H, H-4 and H-6); 5.60 (s, 1H, H-2′); 4.67 (s, 1H, H-9_β_′); 4.66 (s, 1H, H-9_α_′); 3.29 (d, *J* = 7.0 Hz, 2H, H-7′); 2.14 (m, 2H, H-3_β_′ and H-4′); 1.95 (m, 3H, H-3_α_′ and H-6′); 1.74 (m, 1H, H-5_β_′); 1.68 (s, 3H, H-10′); 1.46 (m, 1H, H-5_α_′). ^13^C-NMR (100.6 MHz, CDCl_3_): 156.3 (C-1 and C-3); 155.8 (C-5); 149.7 (C-8′); 137.0 (C-1′); 122.0 (C-2′); 108.5 (C-9′); 103.7 (C-2); 95.7 (C-4 and C-6); 40.9 (C-4′); 30.6 (C-3′ and C-7′); 28.3 (C-5′); 27.5 (C-6′); 20.7 (C-10′). HRMS: M + H ion *m*/*z* 261.1496 (calcd. for C_16_H_21_O_3_, 261.1491).

### 3.3. Cell Lines

The cell lines used in this work were obtained from the American Type Culture Collection (Rockville, MD, USA). They included human prostate cancer cells (PC-3), breast carcinoma cells (MCF-7), human colorectal adenocarcinoma cells (HT-29), human dermal fibroblast cells (HDF) and human colonic epithelial cells (CoN). Cells were grown by the procedure previously described in reference [48].

### 3.4. In Vitro Assays for Cellular Viability

The sulforhodamine B assay was assessed by the procedure previously described in reference [49]. Daunorubicin and dunnione were used as positive controls.

### 3.5. Hoechst 33342 Assay

Morphological changes in the nuclear chromatin of cells undergoing apoptosis were revealed by a nuclear fluorescent dye, Hoescht 33342 (Sigma-Aldrich, Santiago, Chile) [40]. Briefly, on 24-well chamber slides, 1 × 10^4^ cells/mL MCF-7, PC-3 and HDF were cultured and exposed to 25 μM compounds for 48 h. The control group was also exposed to ethanol 1%. After treatments the cells were washed twice with phosphate buffer solution, fixed with 3.7% formaldehyde and washed again with phosphate buffer solution. Following the addition of 1 µM Hoechst 33342, the cells were incubated in a dark room at room temperature for 30 min. After being washed, the cells were examined under an immunofluorescence microscope (Olympus IX 81 model inverted microscope, Olympus, Tokyo, Japan).

### 3.6. Analysis of Mitochondrial Membrane Permeability

Rhodamine 123, a cationic voltage-sensitive probe that accumulates in mitochondria was used to track down changes in mitochondrial membrane permeability [40]. Exponentially growing cells were incubated with compounds as indicated previously. Cells were labeled with rhodamine 123 (1 μM final concentration) at 37 °C in culture medium for 60 min before terminating the experiment. Cells were washed with ice cold phosphate-buffered saline (PBS) and were detached from the plate, the samples were analyzed by flow cytometry. Data is expressed in percentage of cells without rhodamine 123.

### 3.7. Caspases Activity Assay

The activity of caspases was determined by using the CaspACE™ FITC-VAD-FMK (Promega, Santiago, Chile). Briefly, cells were treated with the analysed compounds (0 and 25 µM) for 48 h. The cells were incubated with CaspACE™ FITC-VAD-FMK in darkness for 20 min at room temperature. Then, the medium was removed and cells were washed twice with PBS. Exposed cells were collected by tripsinization and centrifugation (10 min at 1500× *g*). The supernatant was discarded and the cells were re-suspended in PBS and analyzed by flow cytometry using the filter FL3. Results are expressed as percentage of cells stained with CaspACE™ FITC-VAD-FMK [50].

### 3.8. Statistics

Determinations of in vitro assays were performed in triplicate and the results expressed as mean values ± SD. Statistical significance was defined as *p* < 0.05. To analyze the normality in the distribution of the data, the test “Shapiro-Wilk” was used. While for the statistical analysis of data with no normal distribution, the non-parametric test of “Wilcoxon” with designed range was used.

## 4. Conclusions

Starting from perillyl alcohol and synthetic phenols, new cyclodiprenyl phenols **2**–**7** were synthesized and were tested as potential antiproliferative agents against different cancer cell lines. Among all the compounds tested, compound **2** showed a strong antiproliferative activity against breast and prostate cancer cell cultures, while **3** and **4** presented a moderate effect. The results suggest that new dihydroxylated products present better properties than perillyl alcohol. Additional studies are needed to confirm the therapeutical potential of the new cyclodiprenyl phenols as well as to assess their mechanisms of action.

## Figures and Tables

**Table 1:** In vitro cytotoxic activity of natural compound **1** and derivatives **2**–**7**.

Compound	IC_50_ (µM)				
	MCF-7	PC-3	HT-29	CoN	HDF
**1**	>100	>100	>100	>100	>100
**2**	25.9 ± 0.1	12.2 ± 0.7	45.1 ± 0.2	>100	>100
**3**	53.7 ± 0.4	54.5 ± 0.5	>100	>100	>100
**4**	44.3 ± 0.7	79.0 ± 0.2	>100	>100	>100
**5**	37.0 ± 0.1	20.5 ± 0.4	>100	>100	>100
**6**	>100	>100	>100	>100	>100
**7**	>100	>100	>100	>100	>100
Dunnione	14.56 ± 0.04	26.51 ± 0.05	30.32 ± 0.05	24.07 ± 0.55	27.03 ± 0.65
Daunorubicin	0.21 ± 0.01	0.39 ± 0.06	14.7 ± 0.9	-	14.09 ± 0.45

**Table 2:** Percentage of condensed and/or fragmented nuclei after treatment with compounds **2**–**5**.

Compound	MCF-7	PC-3	HDF
**2**	29.4 ± 4.3 **	31.4 ± 3.3 **	8.7 ± 1.4
**3**	16.8 ± 3.1 *	14.5 ± 3.1 *	6.8 ± 1.6
**4**	17.2 ± 2.9 *	13.8 ± 2.1 *	5.6 ± 1.1
**5**	7.3 ± 1.4	6.0 ± 0.3	7.9 ± 1.2
Control	6.7 ± 1.5	8.6 ± 1.0	6.0 ± 1.1

Values are mean ± S.D. (*n* = 3); * *p* < 0.05; ** *p* < 0.01, significantly different from the control-treated cells.

**Table 3:** Percentage of cells without rhodamine-123 after treatment with compounds **2**–**4** on MCF-7 and PC-3-treated cells.

Compound	MCF-7	PC-3
**2**	69.4 ± 4.3 **	41.4 ± 4.8 *
**3**	28.9 ± 2.4 *	29.5 ± 3.7 *
**4**	34.2 ± 4.9 *	26.5 ± 3.8 *
Control	23.0 ± 2.1	15.6 ± 4.0

Values are mean ± S.D. (*n* = 3); * *p* < 0.05; ** *p* < 0.01, significantly different from the control-treated cells.

**Table 4:** Percentage of cells with caspases active on MCF-7 and PC-3-treated cells.

Compound	MCF-7	PC-3
**2**	32.4 ± 4.0 **	23.4 ± 3.0 *
**3**	15.1 ± 3.2 *	16.5 ± 2.3 *
**4**	21.4 ± 2.9 *	19.1 ± 2.8 *
Control	9.1 ± 2.1	8.9 ± 2.1

Values are mean ± S.D. (*n* = 3); * *p* < 0.05; ** *p* < 0.01, significantly different from the control-treated cells.

## Supplementary Materials

The following are available online. Figure S1: Nuclear magnetic resonance spectra: compounds **2**–**7**, Figure S2: High-resolution mass spectra: compounds **2**–**7**, Figure S3: Infrared spectra: compounds **2**–**7**.
