# Supplementary material for: Synthesis and Antiproliferative Activity of New Cyclodiprenyl Phenols against Select Cancer Cell Lines
Source: Molecules. 2018 Sep 12;23(9):2323. doi: 10.3390/molecules23092323 (PMC6225466; doi:10.3390/molecules23092323)
Supplement: Supplementary file 1 [file molecules-23-02323-s001.pdf]

SUPPORTING INFORMATION

Synthesis and Antiproliferative Activity of New Cyclodiprenyl Phenols against Select Cancer Cell Lines

Bastián Said [1], Iván Montenegro [2], Manuel Valenzuela [3], Yusser Olguín [4], Nelson Caro [5], Enrique Werner [6], Patricio Godoy [7], Joan Villena [8,*] and Alejandro Madrid [9,*]

[1] Departamento de Química, Universidad Técnica Federico Santa María, Av. Santa María 6400, Vitacura 7630000, Santiago, Chile; bastian.said@usm.cl
[2] Escuela de Obstetricia y Puericultura, Facultad de medicina, Campus de la Salud, Universidad de Valparaíso, Angamos 655, Reñaca, Viña del Mar 2520000, Chile; E-Mail: ivan.montenegro@uv.cl
[3] Laboratorio de Microbiología Celular, Instituto de Investigación e Innovación en Salud, Facultad de Ciencias de la Salud, Universidad Central de Chile, Santiago 8320000, Chile; E-Mail: manuel.valenzuela@ucentral.cl
[4] Center for Integrative Medicine and Innovative Science (CIMIS), Facultad de Medicina, Universidad Andrés Bello, Santiago 8320000, Chile; yusser.olguin@unab.cl
[5] Centro de Investigación Australbiotech, Universidad Santo Tomás, Avda. Ejército 146, Santiago 8320000, Chile; ncaro@australbiotech.cl
[6] Departamento De Ciencias Básicas, Campus Fernando May Universidad del Biobío. Avda. Andrés Bello s/n casilla 447, Chillán 3780000, Chile; ewerner@ubiobio.cl
[7] Instituto de Microbiología Clínica, Facultad de Medicina, Universidad Austral de Chile, Los Laureles s/n, Isla Teja, Valdivia 5090000, Chile; patricio.godoy@uach.cl
[8] Centro de Investigaciones Biomedicas (CIB), Facultad de Medicina, Campus de la Salud, Universidad de Valparaíso, Angamos 655, Reñaca, Viña del Mar 2520000, Chile; E-Mail: juan.villena@uv.cl
[9] Laboratorio de Productos Naturales y Síntesis Orgánica, Departamento de Química, Facultad de Ciencias Naturales y Exactas, Universidad de Playa Ancha, Avda. Leopoldo Carvallo 270, Playa Ancha, Valparaíso 2340000, Chile; E-Mail: alejandro.madrid@upla.cl
* Correspondence: alejandro.madrid@upla.cl; Tel.: +56-032-250-0526

Supplementary Material

S.1 Nuclear magnetic resonance spectra

S.2 High-resolution mass spectra

S.3 Infrared spectra

S.1 Nuclear magnetic resonance spectra

Compound **2**

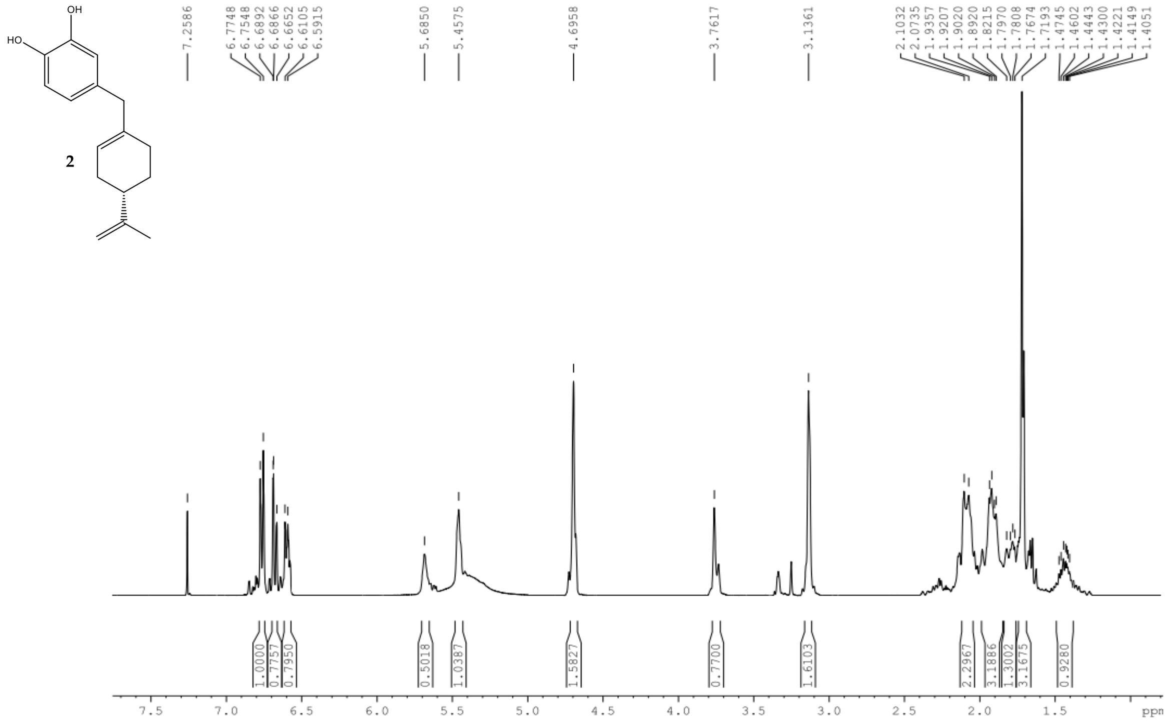

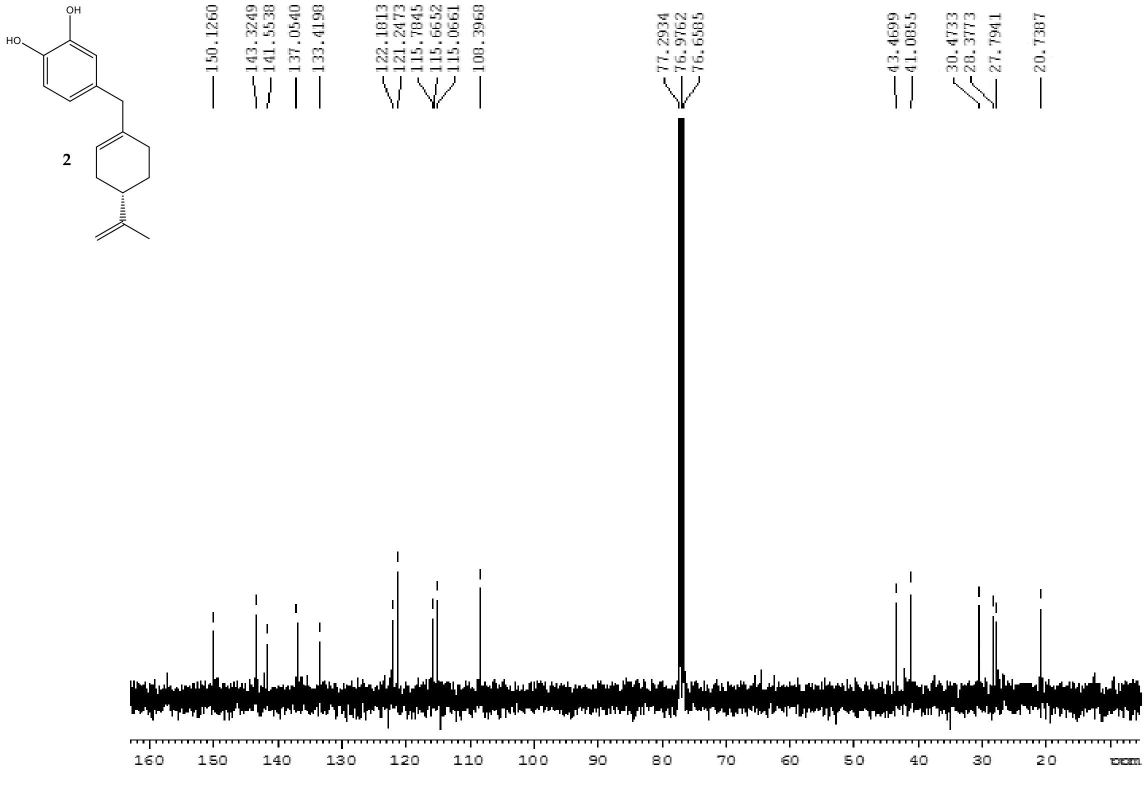

Compound **3**

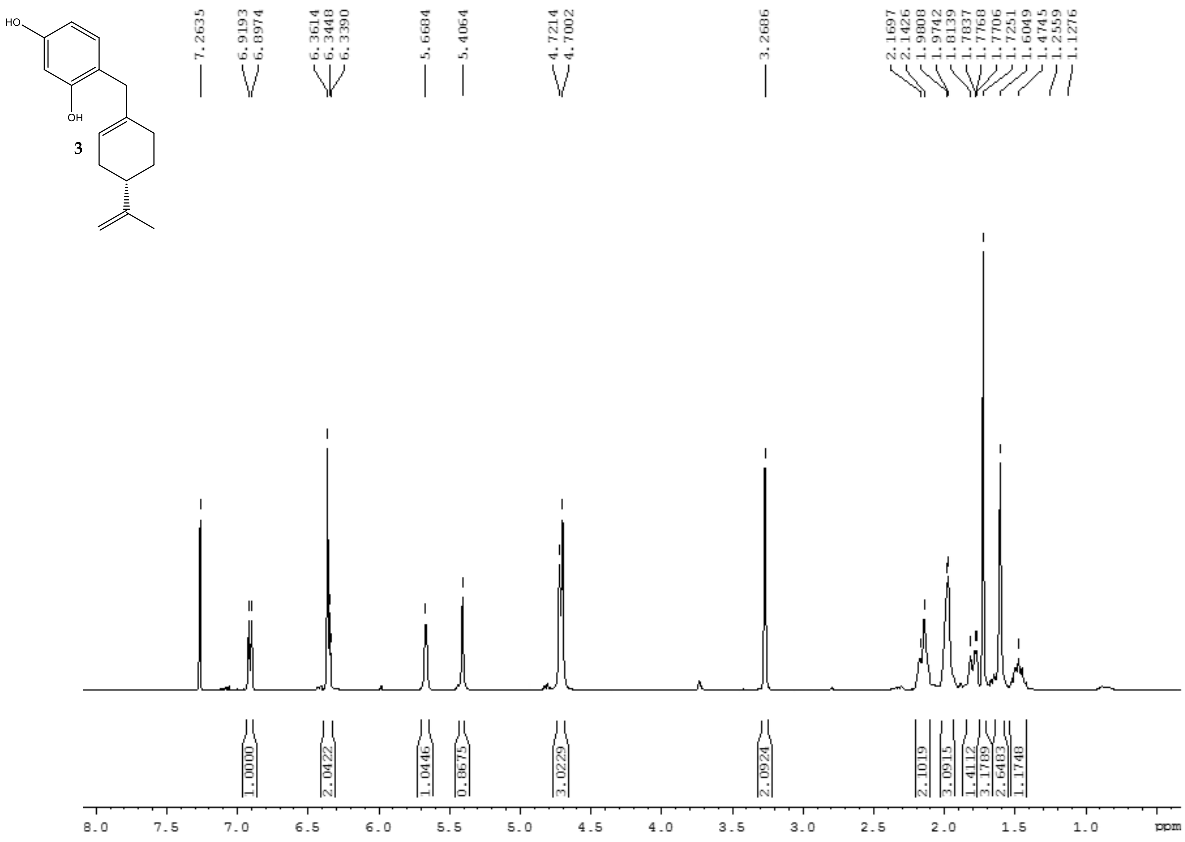

HO
OH
3
7.2635
6.9193
6.8974
6.3614
6.3448
6.3390
5.6684
5.4064
4.7214
4.7002
3.2686
2.1697
2.1426
1.9808
1.9742
1.8139
1.7837
1.7768
1.7706
1.7251
1.6049
1.4745
1.2559
1.1276
1.0000
2.0422
1.0446
0.8675
3.0229
2.0924
2.1019
3.0915
1.4112
3.1789
2.6483
1.1748
8.0 7.5 7.0 6.5 6.0 5.5 5.0 4.5 4.0 3.5 3.0 2.5 2.0 1.5 1.0 ppm

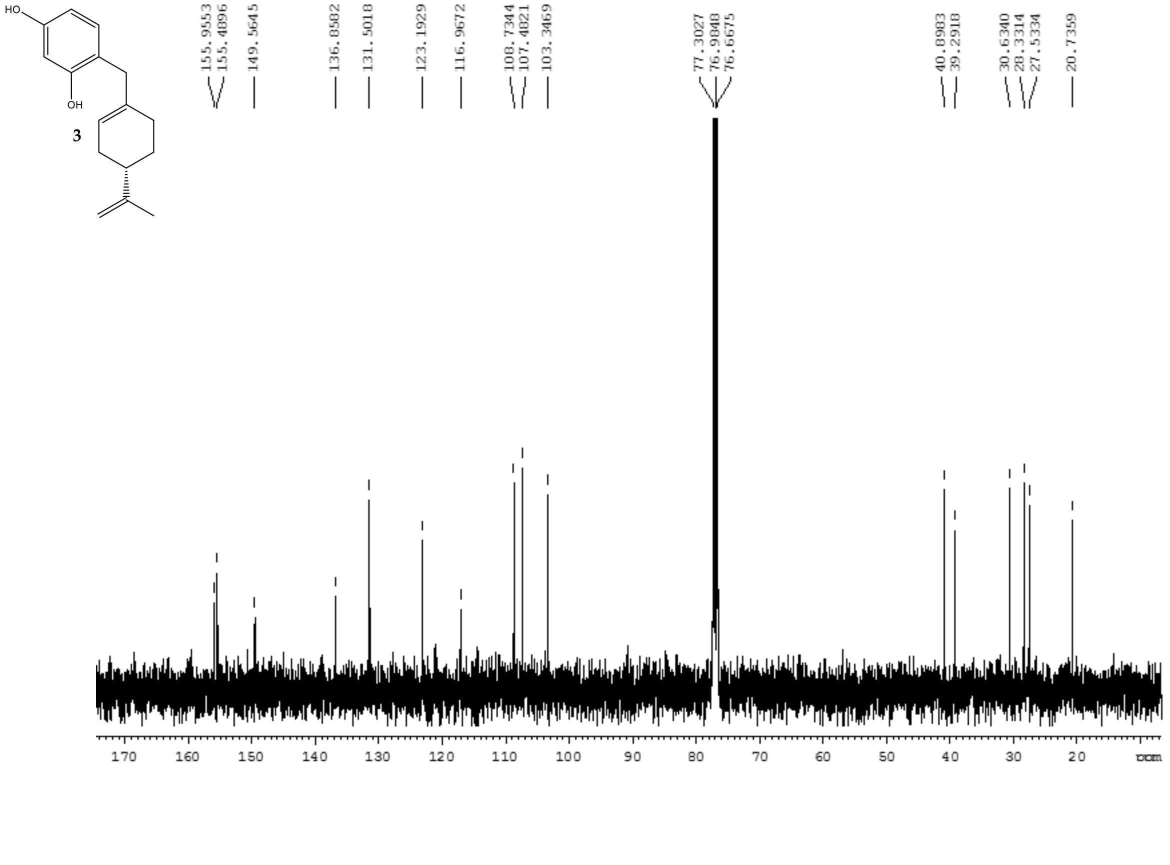

HO
OH
3
155.9553
155.4896
149.5645
136.8582
131.5018
123.1929
116.9672
108.7344
107.4821
103.3469
77.3027
76.9848
76.6675
40.8983
39.2918
30.6340
28.3314
27.5334
20.7359
170 160 150 140 130 120 110 100 90 80 70 60 50 40 30 20 ppm

Compound **4**

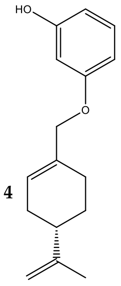

4

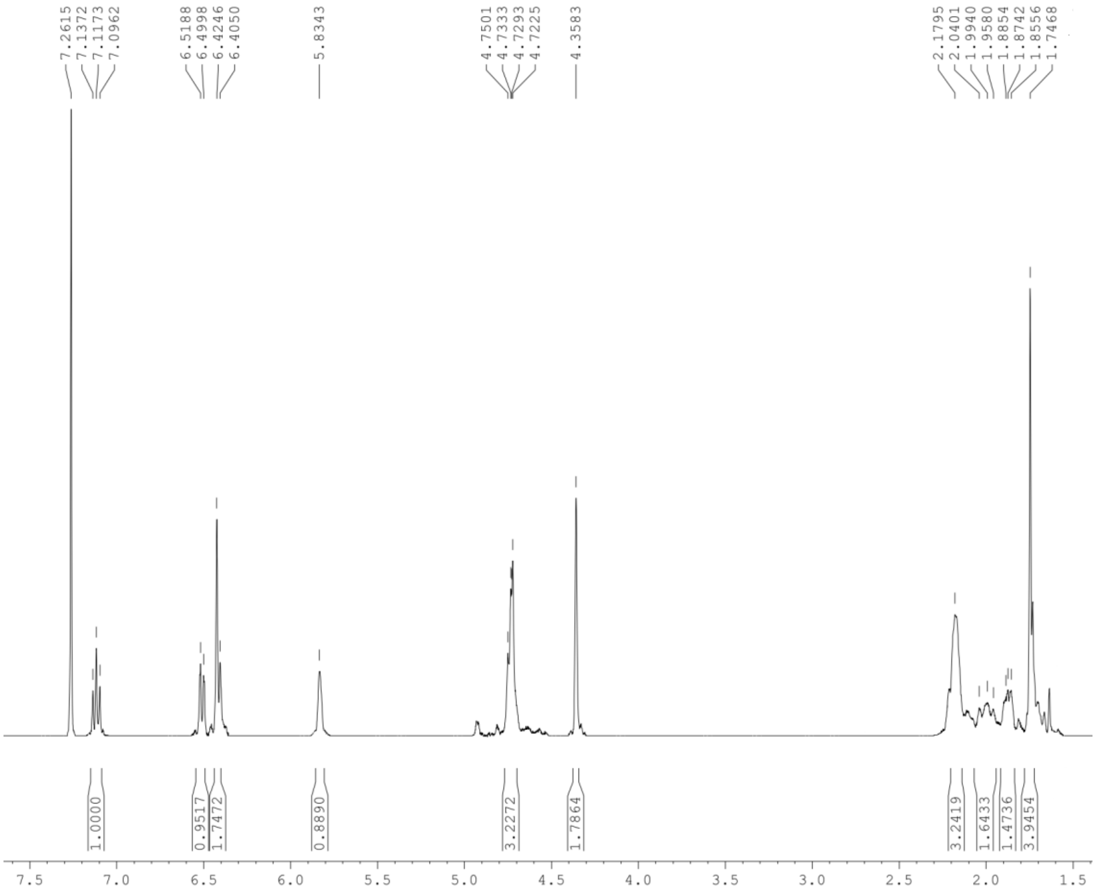

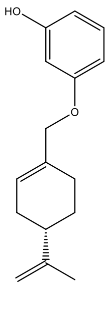

4

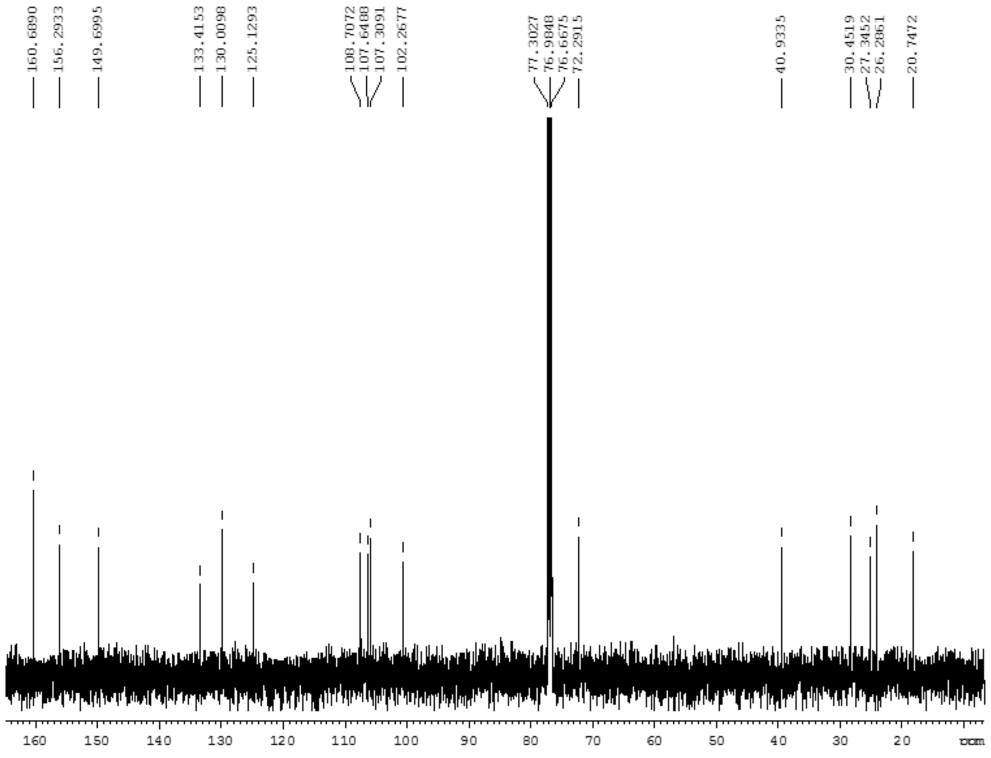

Compound **5**

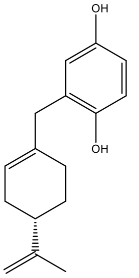

5

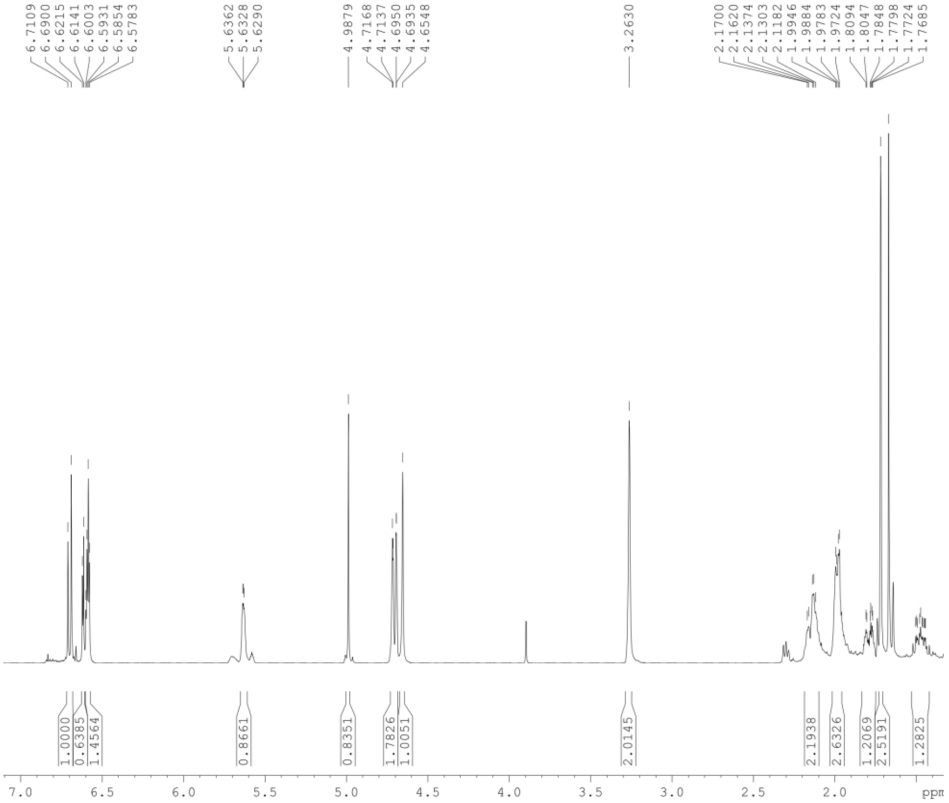

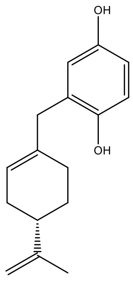

5

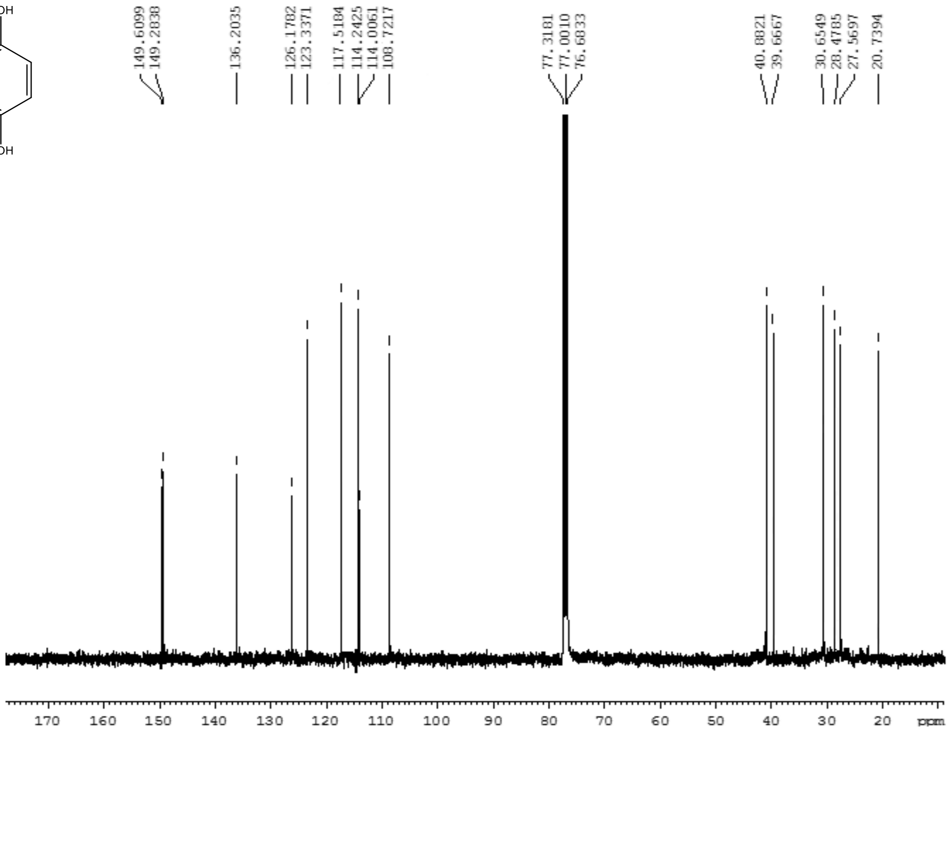

Compound **6**

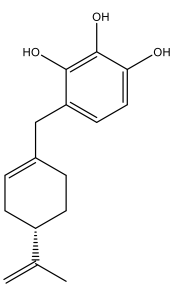

6

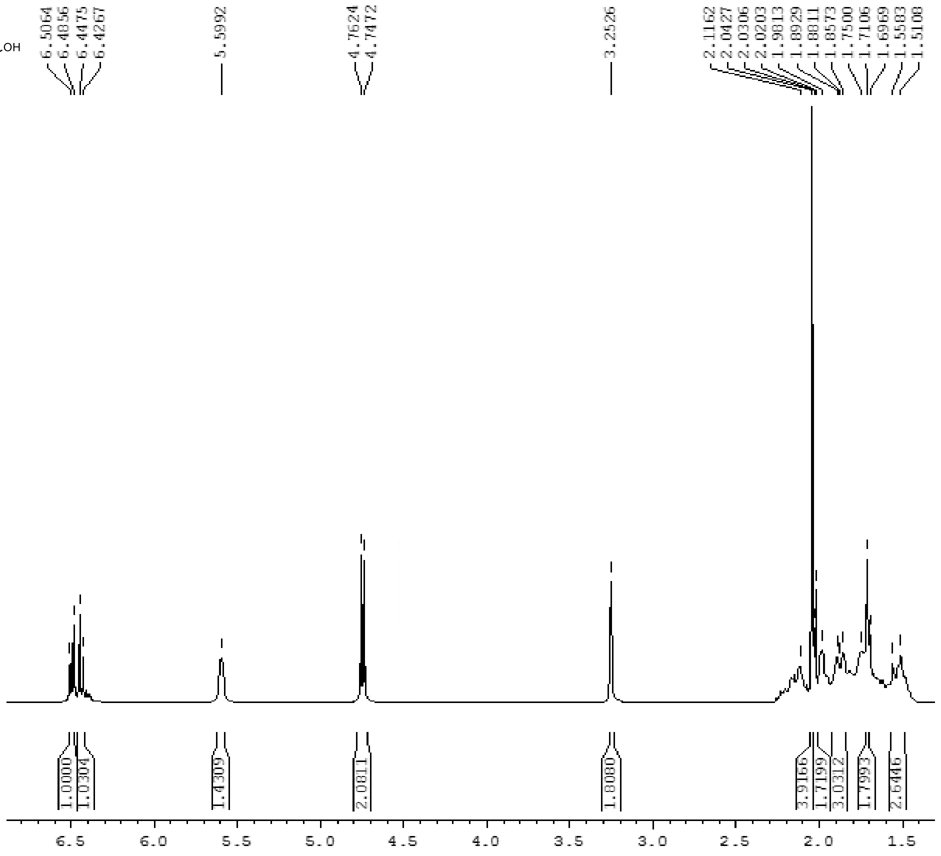

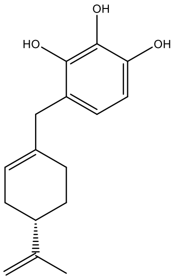

6

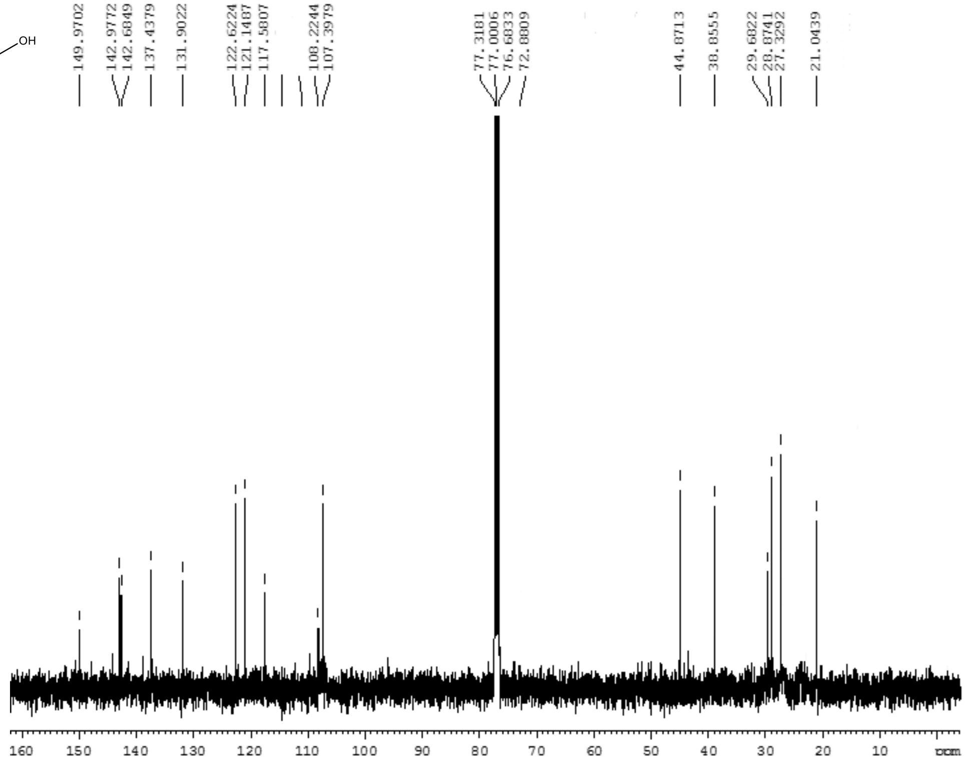

Compound **7**

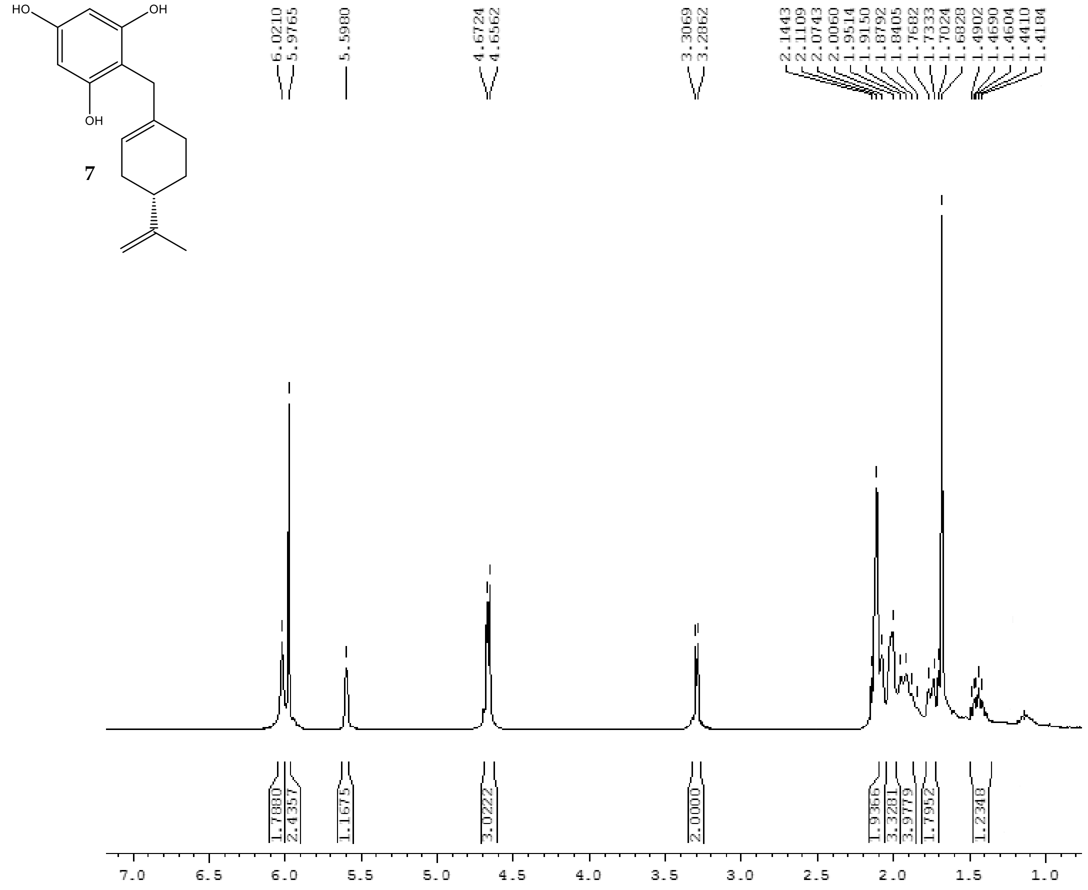
HO
OH
OH
7
6.0210
5.9765
5.5980
4.6724
4.6562
3.3069
3.2862
2.1443
2.1109
2.0743
2.0060
1.9514
1.9150
1.8792
1.8405
1.7682
1.7333
1.7024
1.6828
1.4902
1.4690
1.4604
1.4410
1.4184
1.7880
2.4357
1.1675
3.0222
2.0000
1.9366
3.3281
3.9779
1.7952
1.2348
7.0 6.5 6.0 5.5 5.0 4.5 4.0 3.5 3.0 2.5 2.0 1.5 1.0

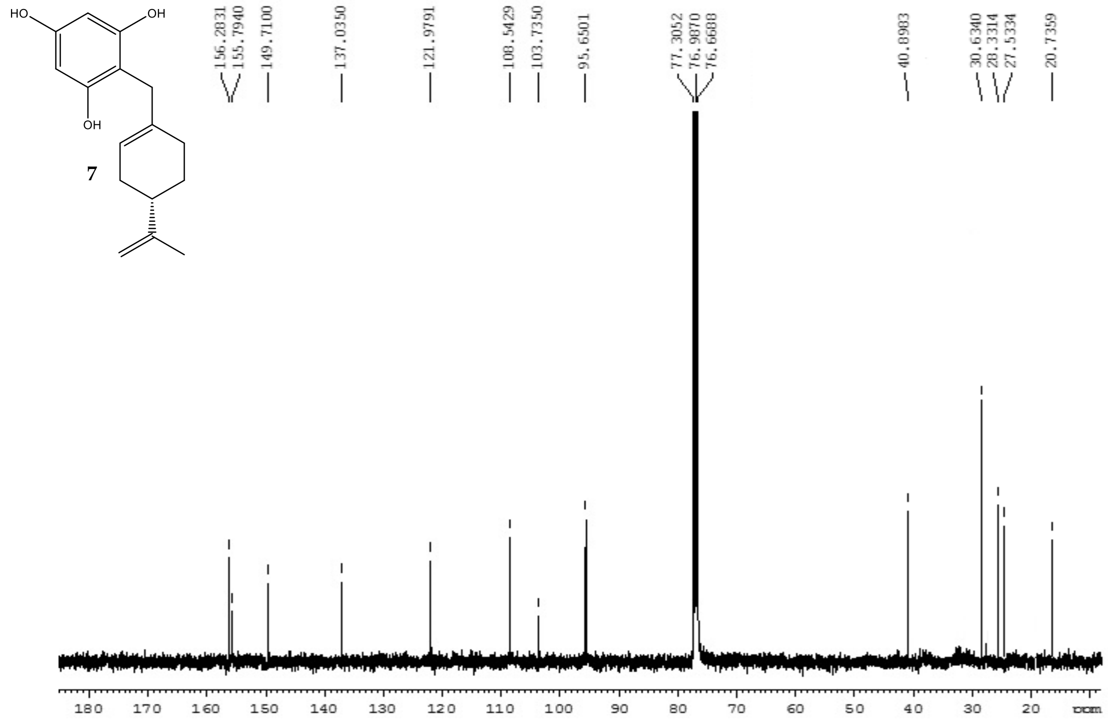
HO
OH
OH
7
156.2831
155.7940
149.7100
137.0350
121.9791
108.5429
103.7350
95.6501
77.3052
76.9870
76.6688
40.8983
30.6340
28.3314
27.5334
20.7359
180 170 160 150 140 130 120 110 100 90 80 70 60 50 40 30 20 ppm

S.2 High resolution mass spectrometry

Compound **2**

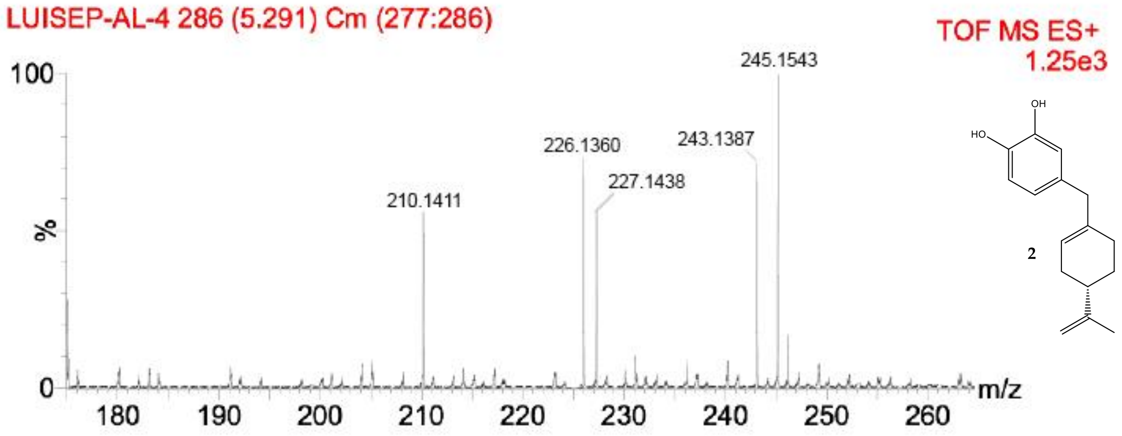

Compound **3**

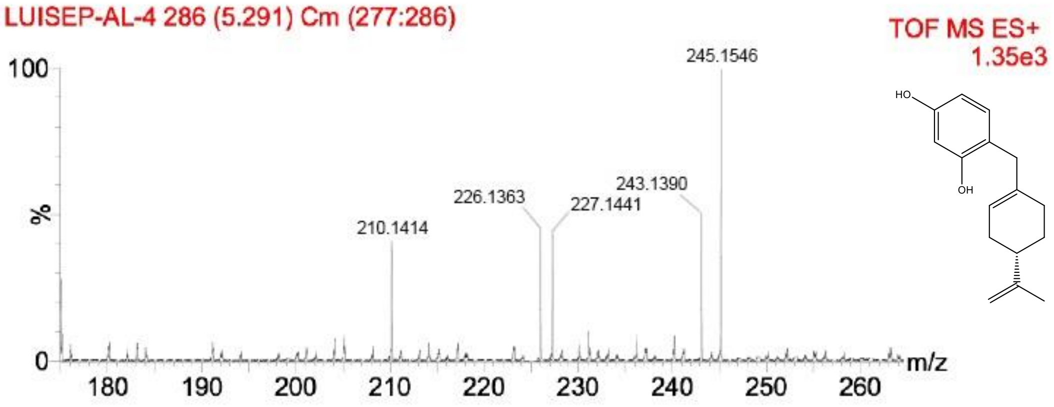

Compound **4**

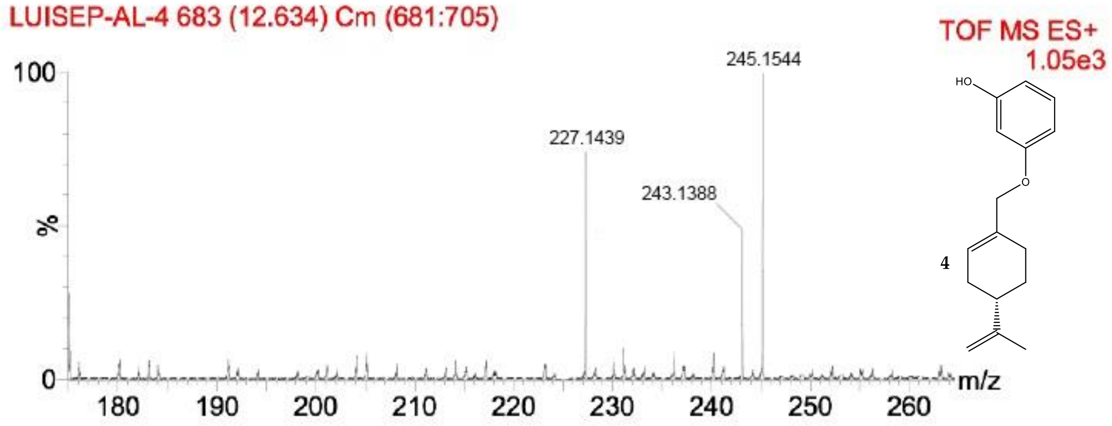

Compound **5**

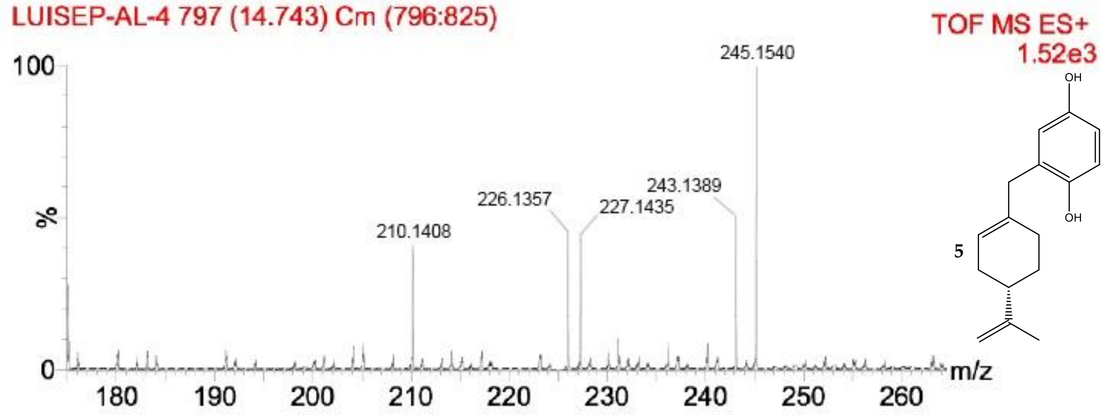

Compound **6**

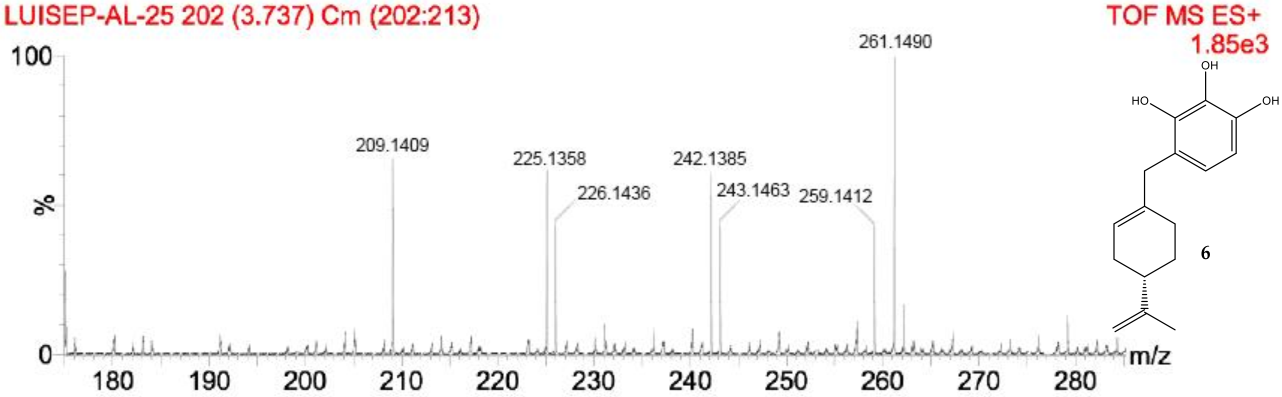

LUISEP-AL-25 202 (3.737) Cm (202:213)
TOF MS ES+
1.85e3
261.1490
209.1409
225.1358
226.1436
242.1385
243.1463
259.1412
100
%
0
m/z
180
190
200
210
220
230
240
250
260
270
280
OH
HO
OH
6

Compound **7**

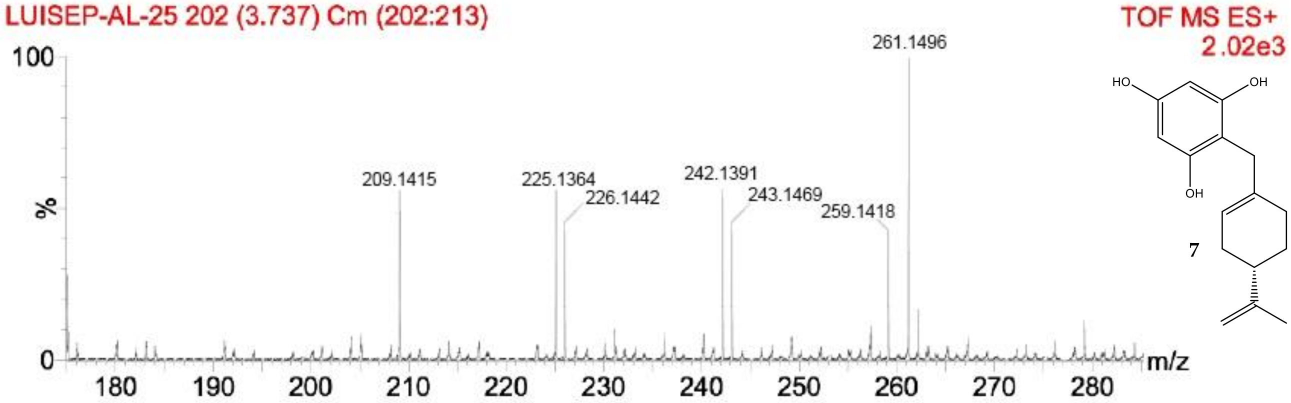

LUISEP-AL-25 202 (3.737) Cm (202:213)
TOF MS ES+
2.02e3
261.1496
209.1415
225.1364
226.1442
242.1391
243.1469
259.1418
100
%
0
m/z
180
190
200
210
220
230
240
250
260
270
280
HO
OH
OH
7

S.3 Infrared spectra

Compound **2**

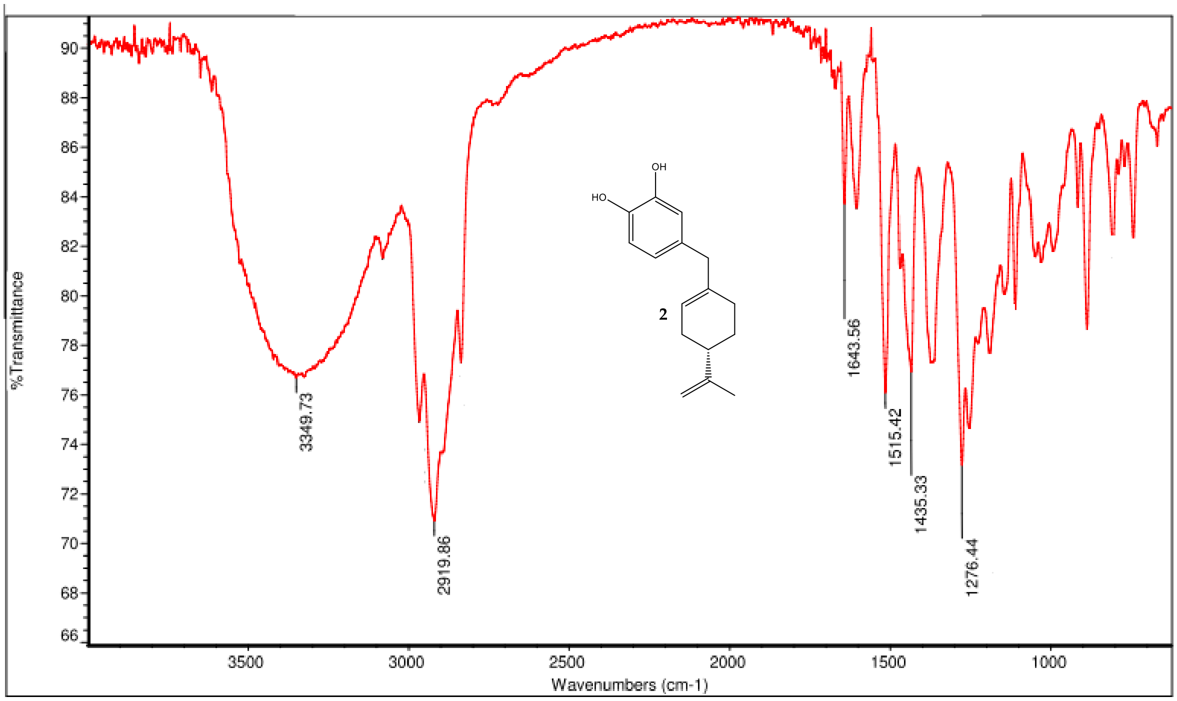

Compound **3**

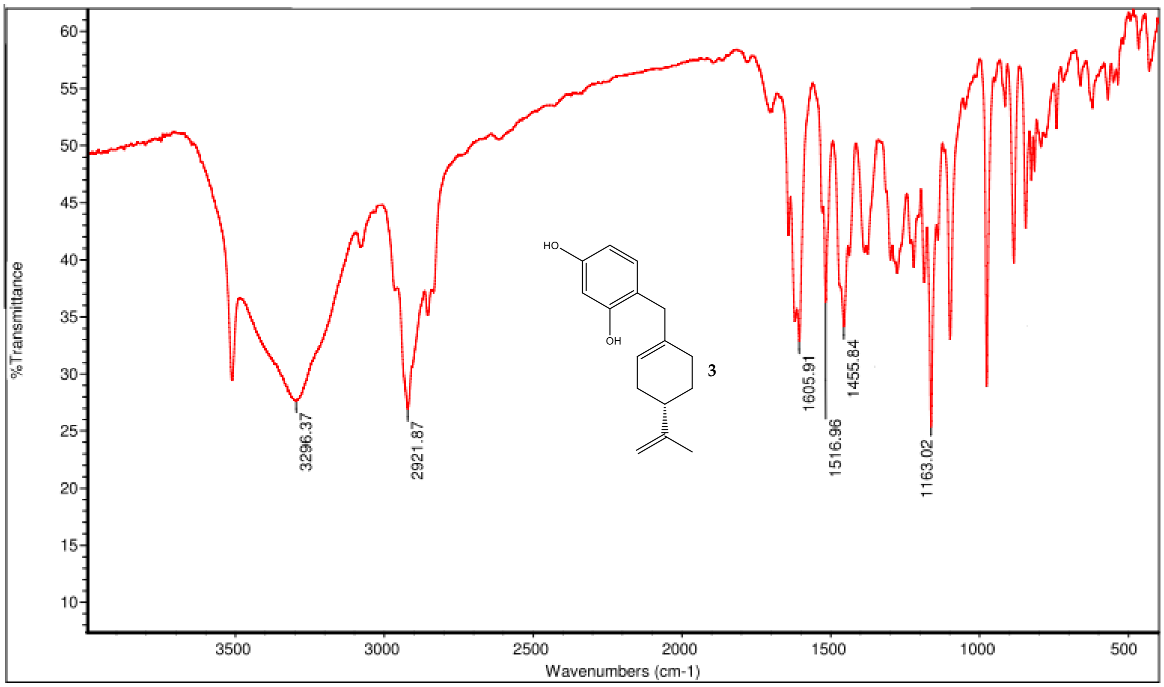

%Transmittance
60
55
50
45
40
35
30
25
20
15
10
3500
3000
2500
2000
1500
1000
500
Wavenumbers (cm-1)
3296.37
2921.87
1605.91
1516.96
1455.84
1163.02
HO
OH
3

Compound **4**

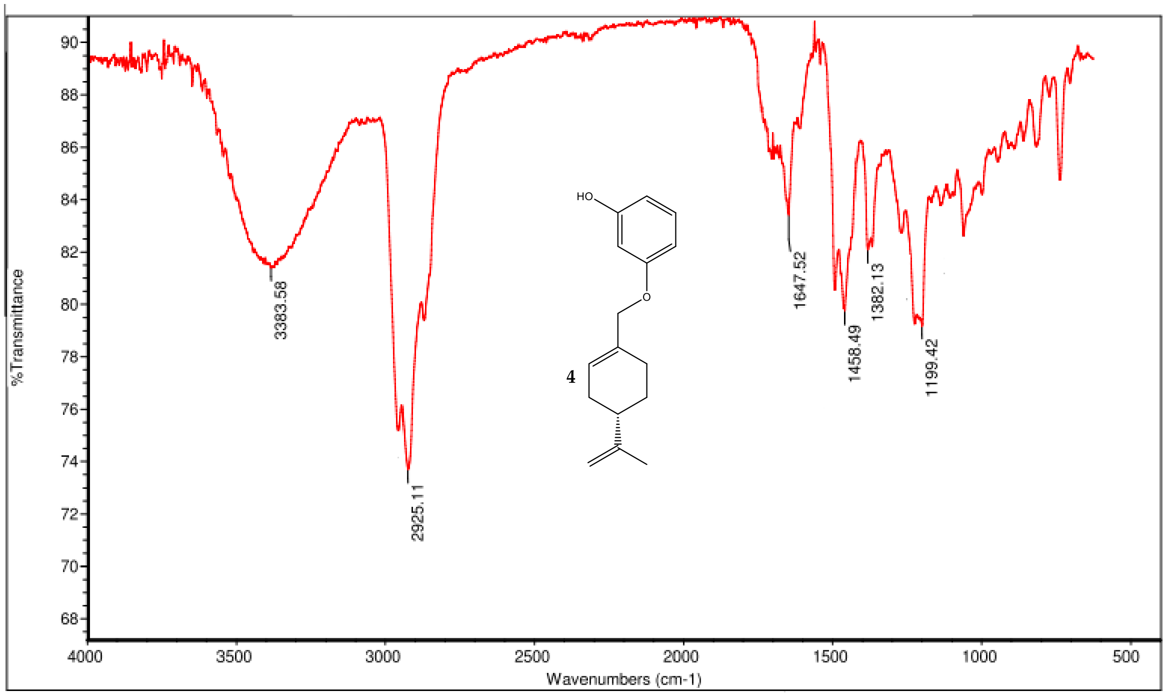

%Transmittance
Wavenumbers (cm-1)
3383.58
2925.11
1647.52
1458.49
1382.13
1199.42
HO
O
4

Compound **5**

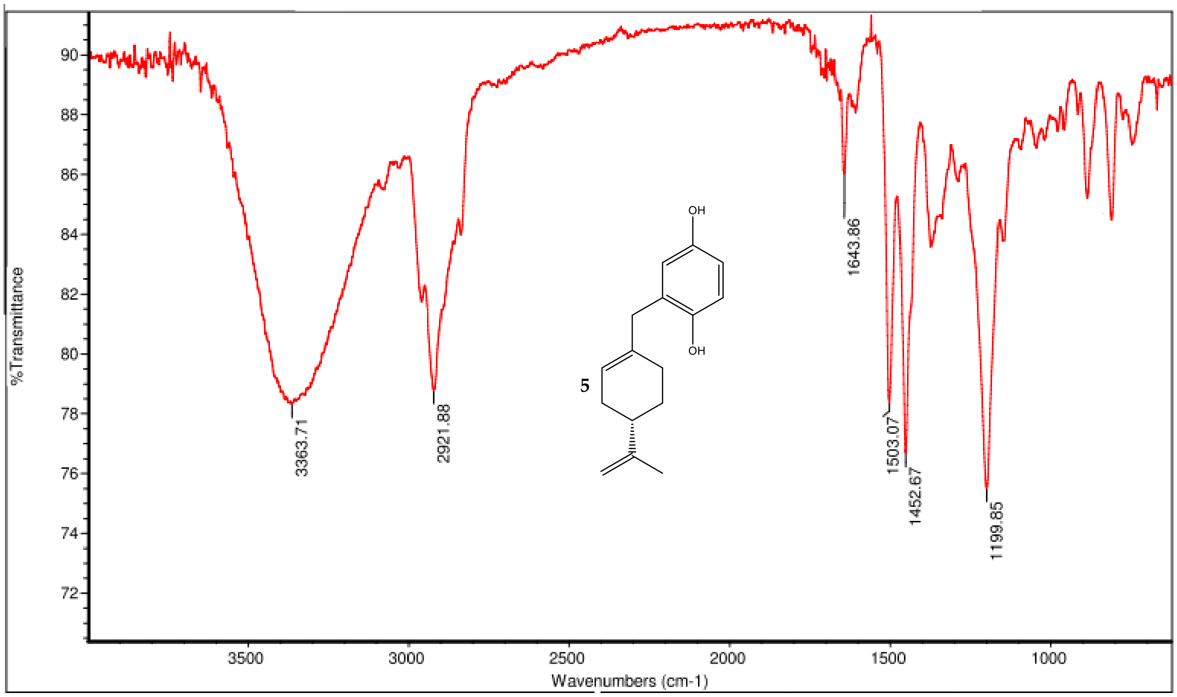

%Transmittance
90
88
86
84
82
80
78
76
74
72
3363.71
2921.88
1643.86
1503.07
1452.67
1199.85
OH
OH
5
3500
3000
2500
2000
1500
1000
Wavenumbers (cm-1)

Compound **6**

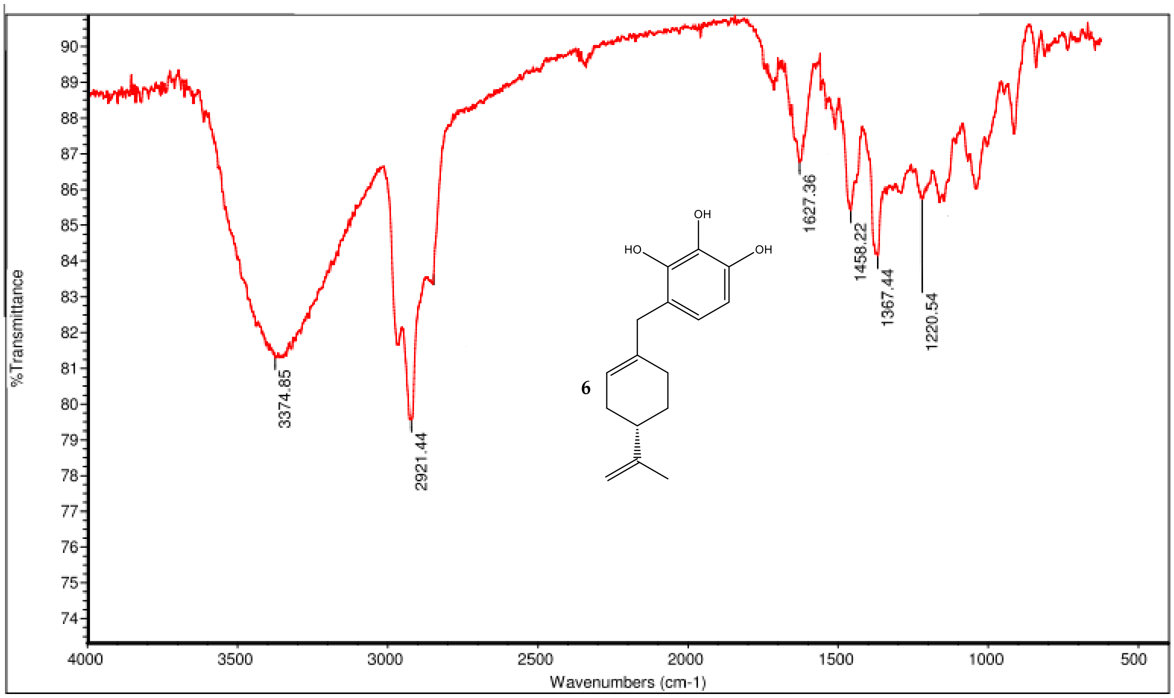

%Transmittance
90
89
88
87
86
85
84
83
82
81
80
79
78
77
76
75
74
4000
3500
3000
2500
2000
1500
1000
500
Wavenumbers (cm-1)
3374.85
2921.44
1627.36
1458.22
1367.44
1220.54
OH
HO
OH
6

Compound **7**

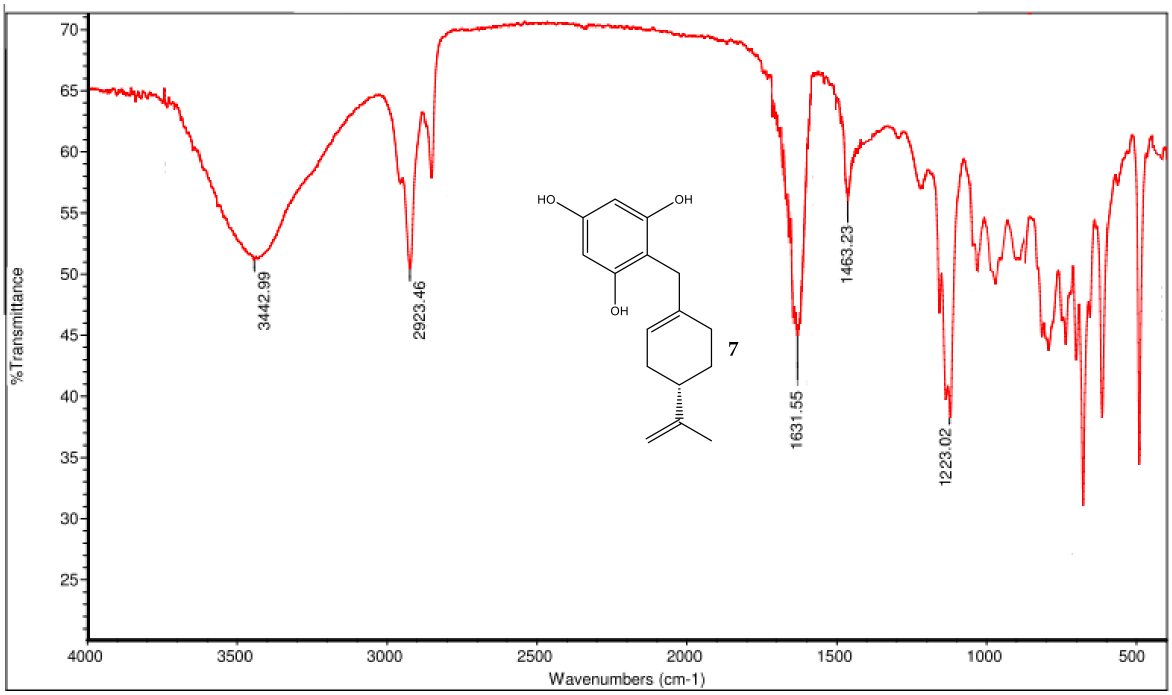

%Transmittance
70
65
60
55
50
45
40
35
30
25
4000
3500
3000
2500
2000
1500
1000
500
Wavenumbers (cm-1)
3442.99
2923.46
1631.55
1463.23
1223.02
HO
OH
OH
7
